# spAttClu: a spatial domain clustering model leveraging spatially weighted graph attention and contrastive learning

**DOI:** 10.1093/bioinformatics/btag384

**Published:** 2026-06-13

**Authors:** Tianjiao Zhang, Ruolan Zhang, Hongfei Zhang, Zhongqian Zhao, Ruihan Wang, Shenghe Li, Yucai Jiang, Binyang Wei, Guohua Wang

**Affiliations:** School of Computer Science and Artificial Intelligence, Northeast Forestry University, Harbin, 150040, China; School of Computer Science and Artificial Intelligence, Northeast Forestry University, Harbin, 150040, China; School of Computer Science and Artificial Intelligence, Northeast Forestry University, Harbin, 150040, China; School of Computer Science and Artificial Intelligence, Northeast Forestry University, Harbin, 150040, China; School of Computer Science and Artificial Intelligence, Northeast Forestry University, Harbin, 150040, China; School of Computer Science and Artificial Intelligence, Northeast Forestry University, Harbin, 150040, China; School of Computer Science and Artificial Intelligence, Northeast Forestry University, Harbin, 150040, China; School of Computer Science and Artificial Intelligence, Northeast Forestry University, Harbin, 150040, China; School of Computer Science and Artificial Intelligence, Northeast Forestry University, Harbin, 150040, China; Faculty of Computing, Harbin Institute of Technology, Harbin, 150001, China

## Abstract

**Motivation:**

The rapid growth of spatial transcriptomics data holds potential for deep understanding of spatial specificity and tissue heterogeneity. Recognizing spatial domains is a fundamental step for deciphering tissue functional architecture and dissecting tissue heterogeneity. However, existing models typically define adjacency relations using static weights, which cannot dynamically adjust neighbor importance based on expression context, thereby limiting the accuracy and robustness of spatial domain recognition.

**Results:**

We propose spAttClu, a clustering model integrating spatially weighted graph attention with contrastive learning. It adaptively learns neighbor contributions in varying contexts through a distance-weighted graph attention mechanism and enhances embedding discriminability via multi-level contrastive learning. spAttClu demonstrates superior clustering performance on the DLPFC dataset. Moreover, it shows cross-platform adaptability and enables vertical/horizontal inte-gration of multiple tissue slices.

## 1 Introduction

In multicellular organisms, cellular function is jointly regulated by transcriptional state and spatial location (Dong and Zhang 2022, Xu *et al.* 2022, 2025). Spatial transcriptomics (ST) technologies enable the concurrent analysis of gene expression and spatial context, providing novel insights into tissue complexity (Moffitt, Lundberg and Heyn 2022, Moses and Pachter 2022, Lu *et al.* 2025, Xie *et al.* 2026). However, the sheer volume of data does not directly reveal biological principles. The core computational step, and a current foundational challenge, is to partition transcriptionally similar and spatially contiguous spots into distinct functional spatial domains to decipher tissue functional units (Wang *et al.* 2025).

To address this challenge, methodologies have evolved from early expression-dependent strategies like K-means (Zhang *et al.* 2025) to those integrating spatial proximity. Existing approaches fall into three categories: (1) Graph Neural Network-based methods, such as STAGATE (Dong and Zhang 2022), which adaptively fuse neighbor information using graph attention networks; (2) Probabilistic-statistical models, like BayesSpace (Zhao *et al.* 2021), which enhance noise robustness by introducing spatial smoothing priors within a Bayesian framework; and (3) Contrastive learning frameworks, such as GraphST (Long *et al.* 2023), which employ graph autoencoders with mutual information maximization to learn discriminative embeddings. Additionally, SpaMask (Min *et al.* 2025) adopts a spatial instance segmentation paradigm. Collectively, these methods have advanced the field of spatial clustering.

However, existing models inadequately adaptively regulate the contribution of neighboring nodes (Moffitt *et al.* 2018) as their static weights struggle to adjust dynamically based on local expression contexts. For instance, spaGCN (Hu *et al.* 2021) and spaGIC (Zhang *et al.* 2024) rely on pre-computed Gaussian static edge weights; the attention computation in GAAEST (Wang *et al.* 2024) is constrained by fixed topology; although spaGT (Bao *et al.* 2025) iteratively optimizes edge embeddings, its adjacency relations still originate from fixed rules. This static weighting hinders models from dynamically enhancing relevant or suppressing irrelevant neighbor contributions in heterogeneous regions, thereby limiting clustering accuracy (Cang *et al.* 2023). Furthermore, existing methods also face challenges in multi-slice integration (Ren *et al.* 2022), handling large-scale data (Li and Zhou, 2022), and cross-platform generalization (Maynard *et al.* 2021).

To address these challenges, we propose spAttClu, a clustering model integrating spatially weighted graph attention with contrastive learning. spAttClu converts physical distances into attention prior weights via a Gaussian kernel function, dynamically adjusts the importance of expression contexts, and enables adaptive modeling of complex tissue regions. Additionally, by jointly optimizing local-global mutual information and spatial continuity loss, spAttClu achieves cross-slice alignment and batch correction. It also employs a parameterized graph construction pipeline to support multi-platform data adaptation and large-scale computation. In benchmarking, spAttClu achieved average ARI and NMI scores exceeding 0.8 across 12 slices of the DLPFC dataset, outperforming mainstream methods. The model also effectively supports multi-slice integrative analysis and demonstrates stable cross-platform generalization on data from platforms like 10x Visium and Stereo-seq. These results indicate that spAttClu is a comprehensive and robust unified framework for single-slice clustering, multi-slice integration, and cross-platform analysis tasks.

## 2 Materials and methods

### 2.1 Datasets

To comprehensively evaluate the performance of spAttClu, this study employed multiple spatial transcriptomics datasets (listed in Table 1 and Supplementary S1, available as supplementary data at *Bioinformatics* online).

**Table 1:** The summary of datasets.

Dataset	Genes	Spots	Domains	Platform	Reference
DLPFC	33 538	3460–4789	5–7	10x Visium	(Maynard *et al.* 2021)
mouse posterior brain	31 053	3353		10x Visium	(Elosua-Bayes *et al.* 2021)
E9.5 mouse embryo	23,015	5913	12	Stereo-seq	(Chen *et al.* 2022)
Mouse olfactory bulb	26 145	10 000	8	Stereo-seq	(Chen *et al.* 2022)
Mouse somatosensory cortex	33	4839	11	osmFISH	(Wang *et al.* 2018)
Mouse hypothalamic preoptic area	155	5557	8	MERFISH	(Moffitt *et al.* 2018)
Mouse Visual Cortex	1207	1020	8	STARmap	(Wang *et al.* 2018)
E10.5 mouse embryo	25 201	18 408	13	Stereo-seq	(Chen *et al.* 2022)
E14.5 mouse embryo	18 566	92 928	16	Stereo-seq	(Chen *et al.* 2022)
simulated dataset	31 493	640 000	9		(Gong *et al.* 2025)

### 2.2 Evaluation metrics

Depending on the availability of ground-truth spatial domain labels, external and internal metrics were used for a holistic assessment. Due to space constraints, only ARI and NMI are detailed herein. Definitions and calculations for other metrics are provided in Supplementary S2, available as supplementary data at *Bioinformatics* online.

Adjusted Rand Index (ARI): The ARI measures the similarity between predicted labels and ground truth labels. It is a similarity measure between two clustering, with values ranging from -1 to 1. A value closer to 1 indicates a higher level of agreement (Wang *et al.* 2025). Its calculation formula is:


$$\mathrm{ARI}=\frac{\sum_{i,j}\left(\frac{n_{ij}}{2}\right)-[\sum_{i}\left(\frac{a_{i}}{2}\right)\sum_{j}\left(\frac{b_{j}}{2}\right)]/\left(\frac{n}{2}\right)}{\left[\sum_{i}(\frac{a_{i}}{2})\;+\;\sum_{j}\left(\frac{b_{j}}{2}\right)\right]/2-[\sum_{i}(\frac{a_{i}}{2})\sum_{j}\left(\frac{b_{j}}{2}\right)]/\left(\frac{n}{2}\right)}$$

where n denotes the total number of samples, $n_{i,j}$ represents the number of samples whose ground truth label belongs to class i and are assigned to cluster j, $a_{i}\;=\;\sum_{j}n_{ij}$ is the number of samples in true class i, and $b_{j}\;=\;\sum_{i}n_{ij}$ is the number of samples in predicted cluster j.

Normalized Mutual Information (NMI): NMI is a standardized measure of mutual information, commonly used to quantify the similarity between predicted labels and ground truth labels. Its value ranges from 0 to 1, where a value closer to 1 indicates a higher level of consistency (Müller-Bötticher *et al.* 2025). Its calculation formula is:


$$\mathrm{NMI}\;=\;\frac{MI(L,U)}{F(H(L),H(U))}$$

Where $MI\;=\;\sum_{i=1}^{N}\sum_{j=1}^{C}p_{i,j}\log\left(\frac{p_{\dot{i},j}}{p_{i},p_{j}}\right)$ calculates the mutual information between L and U, $H(L)\;=\;-\sum_{i=1}^{N}p_{i}\log\left(p_{i}\right)$ and $H(U)=\;-\sum_{j=1}^{C}p_{i}\log\left(p_{i}\right)$ represent the entropy of the label vectors L and U, respectively. $F(x_{1},x_{2})$ can be the max, min, or mean function; here we select the mean function.

### 2.3 Spatially weighted Gaussian Kernel attention module

**Figure 1:**
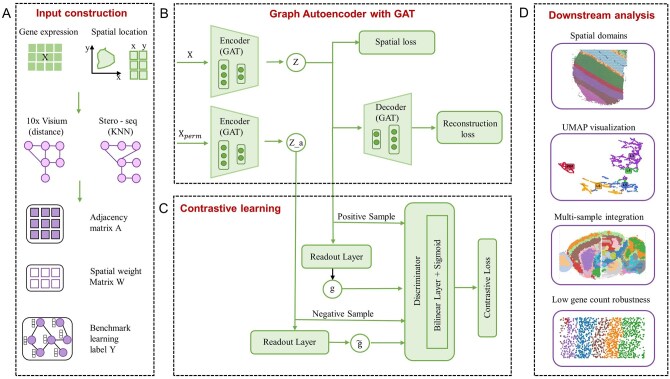
Overview of the spAttClu framework. (A) Input gene expression and spatial coordinates are used to adaptively construct an adjacency graph, generating spatial weights via a Gaussian kernel. (B) The graph attention encoder integrates spatial priors to learn representations, while the decoder reconstructs features and dynamically adjusts expression context importance. (C) Representation optimization via contrastive learning. (D) Downstream analysis.

spAttClu is designed to achieve high-accuracy clustering of spatial transcriptomics data and address its associated challenges. Its architecture consists of two core modules: a spatially weighted Gaussian kernel attention module and a contrastive learning with mutual information maximization module. During data preprocessing, gene expression is first normalized and highly variable genes are selected (Hao *et al.* 2021). An adaptive strategy then constructs a spatial adjacency graph using either K-nearest neighbors or a distance threshold, based on data resolution (neighborhood sizes for different platforms are in Supplementary S3, available as supplementary data at *Bioinformatics* online). Procedures for handling large-scale datasets and integrating multiple sections are detailed in Supplementary S4, available as supplementary data at *Bioinformatics* online. The preprocessing module outputs standardized features X, an adjacency matrix A, a static weight matrix W, and contrastive labels Y, packaged as an Anndata object for subsequent model input. The model structure is shown in Fig. 1.

The spatially weighted Gaussian kernel attention module, a core component of spAttClu, jointly weighs spatial proximity and gene expression similarity to dynamically adjust the importance of expression context (Wang *et al.* 2025).Specifically, this module takes the node gene expression feature matrix $X\in R^{N\times D}$and the spatial adjacency matrix $A\in R^{N\times N}$ as input, where N denotes the number of spatial spots and D represents the feature dimension. First, a linear transformation is applied to the input features: H=XW, where W is a trainable weight matrix. Subsequently, the raw attention coefficients between nodes are computed. In the standard GAT, the attention coefficient is derived from the dot product of the feature projections: $e_{ij}=\mathrm{LeakyReLU}(a^{T}[H_{i}||H_{j}])$, where a is the attention parameter vector. To incorporate spatial priors, this module constructs a distance matrix based on the Euclidean distance between spots and transforms it into a spatial weight matrix using the Gaussian kernel function. The formula is as follows:


$$w_{ij}\;=\;\;\text{exp}(-\frac{d_{ij}^{2}}{2\sigma^{2}})$$

Where $d_{ij}$ denotes the Euclidean distance between spots i and j, and σ represents the median of the non-zero elements in the distance matrix, controlling the rate at which the weight decays with distance. This weight matrix is incorporated as prior knowledge into the computation of the attention coefficients (Han and De Oliveira 2025). This data-driven scheme ensures that σ adapts to the inherent spatial resolution of different datasets, thereby providing a robust and unbiased spatial prior. We select the Gaussian kernel for its smooth decay property, aligning with the spatial transcriptomics continuity prior that closer distance implies higher expression similarity (Svensson *et al.* 2018). Compared with Laplace or uniform kernels, the Gaussian kernel adaptively and analytically controls spatial scale via bandwidth σ. We systematically validated σ robustness in Supplementary S5, available as supplementary data at *Bioinformatics* online indicating a reasonable bias-variance trade-off. The weight is multiplied with the raw attention coefficient: ${\tilde{e}}_{ij}=e_{ij}.\;w_{ij}$, thereby enhancing the attention weights for proximal spot pairs. Subsequently, the masked attention mechanism retains only the adjacency relationships: $\alpha_{ij}={\mathrm{softmax}}_{j}({\tilde{e}}_{ij}).\;A_{ij}$. This design ensures the model considers both gene expression similarity and spatial proximity when computing attention, thereby more accurately capturing the biological associations within the local spatial context. The final output is the weighted feature sum: $Z_{i}=\sum_{j\;\in \;N(i)}\alpha_{ij}H_{j}$. The output of this module is a low-dimensional embedding representation for each spot. Furthermore, comparison with other spatial weighting strategies (Supplementary S6, available as supplementary data at *Bioinformatics* online) validates the effectiveness of the spAttClu spatial weighting strategy.

### 2.4 Contrastive learning and mutual information maximization module

spAttClu employs a contrastive learning module based on Deep Graph Infomax (DGI) (Park *et al.* 2020), which learns batch-invariant representations by maximizing mutual information between local nodes and the global section context. This implicitly corrects technical variation and enables cross-section spatial domain alignment (Liu *et al.* 2024). First, a global graph-level summary vector $g=\mathrm{Readout}(Z)=\frac{1}{N}\sum_{i=1}^{N}z_{i}$ is generated via the AvgReadout module, where $z_{i}$ denotes the node embedding features and N is the number of nodes. This summary vector represents the contextual information of the entire slice. Subsequently, a bilinear discriminator $D(z_{i},g)=\sigma (z_{i}^{T}Wg)$ distinguishes positive sample pairs (node embedding and the true graph summary) from negative sample pairs (node embedding and a negative sample summary $\tilde{g}$ generated via feature permutation). Here, W is a learnable parameter matrix and σ is the sigmoid activation function. Negative sample features ${z\_a}_{i}$ are obtained by globally shuffling the original node feature matrix row-wise (Lu *et al.* 2025). The discriminator loss employs the binary cross-entropy loss:


$$L_{sl}\;=-\;\frac{1}{N}\sum_{i=1}^{N}\left[\mathrm{logD}(z_{i},g)+\log(1-D({z\_a}_{i},\tilde{g}))\right]$$

Where ${z\_a}_{i}$ represents the negative sample node embeddings. This loss function enables the encoder to learn discriminative features that are invariant to noise by maximizing the mutual information between local nodes and the global graph summary. The module takes the original gene expression feature matrix $X\;\in \;R^{N\times D}$ and its permuted version $X_{\mathrm{perm}}$ as input, and outputs the contrastive loss scalar value and the node embedding matrix $Z\;\in \;R^{N\times D}$. The final embedding representation serves as the input for downstream clustering tasks.

### 2.5 Total loss function

The proposed spAttClu model achieves high-accuracy clustering of spatial transcriptomics data by jointly optimizing three loss components: gene expression reconstruction, contrastive learning, and spatial consistency. To simultaneously maintain gene expression characteristics, enhance inter-class discriminability, and conform to tissue spatial continuity, the total loss function is formulated as the following weighted formulation:


$$L\;=\;\alpha .L_{\mathrm{recon}}+\beta .(L_{sl1}+L_{sl2})+\gamma .L_{\mathrm{spatial}}$$

Where $L_{\mathrm{recon}}=\frac{1}{N}\sum_{i=1}^{N}||X_{i}-{\hat{x}}_{i}{\left|\right|}^{2}$ denotes the gene expression reconstruction loss, which employs the mean squared error to quantify the disparity between the input raw features $X_{i}$ and the decoder output ${\hat{x}}_{i}$, thereby ensuring the embedding space retains key gene expression patterns. The hyperparameter α controls its weight. The contrastive loss $L_{sl}$ employs a binary cross-entropy function:


$$L_{sl}\;=-\;\frac{1}{N}\sum_{i=1}^{N}\left[\mathrm{logD}(z_{i},g)+\log(1-D({z\_a}_{i},\tilde{g}))\right]$$

Parameters in the Lsl loss function are introduced in Section 2.4 and omitted here. This loss enhances embedding discriminability by pulling positive sample pairs (original and augmented features) closer together and pushing negative sample pairs apart, with β serving as its weighting coefficient. $L_{sl1}$ maximizes mutual information between real nodes and the global summary, while $L_{sl2}$ enhances robustness of node embeddings against feature permutation noise via perturbation. The spatial consistency loss is defined as:


$$L_{\mathrm{spatial}}\;=\;\frac{1}{\left|\varepsilon \right|}\sum_{(i.j)\in \varepsilon }a_{ij}.\;{\left\|z_{i}-z_{j}\right\|}^{2}$$

Where ε denotes the set of adjacent node pairs, $a_{ij}$ represents the attention weight based on spatial distance, and $z_{i}$ and $z_{j}$ are the embedding vectors of adjacent nodes. This term constrains spatially proximate points to maintain similarity in the latent representation, with the hyperparameter γ modulating its influence strength. Empirically, we set α to 10, β to 1, and γ to 0.1. To validate systematic sensitivity of three parameters α, β, γ, we performed correlation analysis in Supplementary S7, available as supplementary data at *Bioinformatics* online, confirming that this parameter combination achieves the best performance. This loss function takes the highly variable gene expression matrix and spatial adjacency relationships as input, outputs a low-dimensional embedding representation, and effectively supports downstream clustering tasks.

### 2.6 Clustering analysis

This study applies the mclust (Lee *et al.* 2024) clustering method, based on a Gaussian Mixture Model (GMM), to analyze the spatial-aware embedding features (see Supplementary S8, available as supplementary data at *Bioinformatics* online for implementation environment). Hyperparameter settings for the comparative models are detailed in Supplementary S3, available as supplementary data at *Bioinformatics* online. Meanwhile, the main hyperparameters of this model are summarized in Supplementary Table S1, available as supplementary data at *Bioinformatics* online.

## 3 Results

### 3.1 spAttClu significantly enhances clustering accuracy in the human dorsolateral prefrontal cortex dataset

A core challenge in spatial transcriptomics clustering is to deeply integrate spatial information for precise recognition of biologically meaningful spatial domains. This study evaluates model performance on this task using the 10x Visium human dorsolateral prefrontal cortex (DLPFC) dataset with detailed laminar annotations.

**Figure 2:**
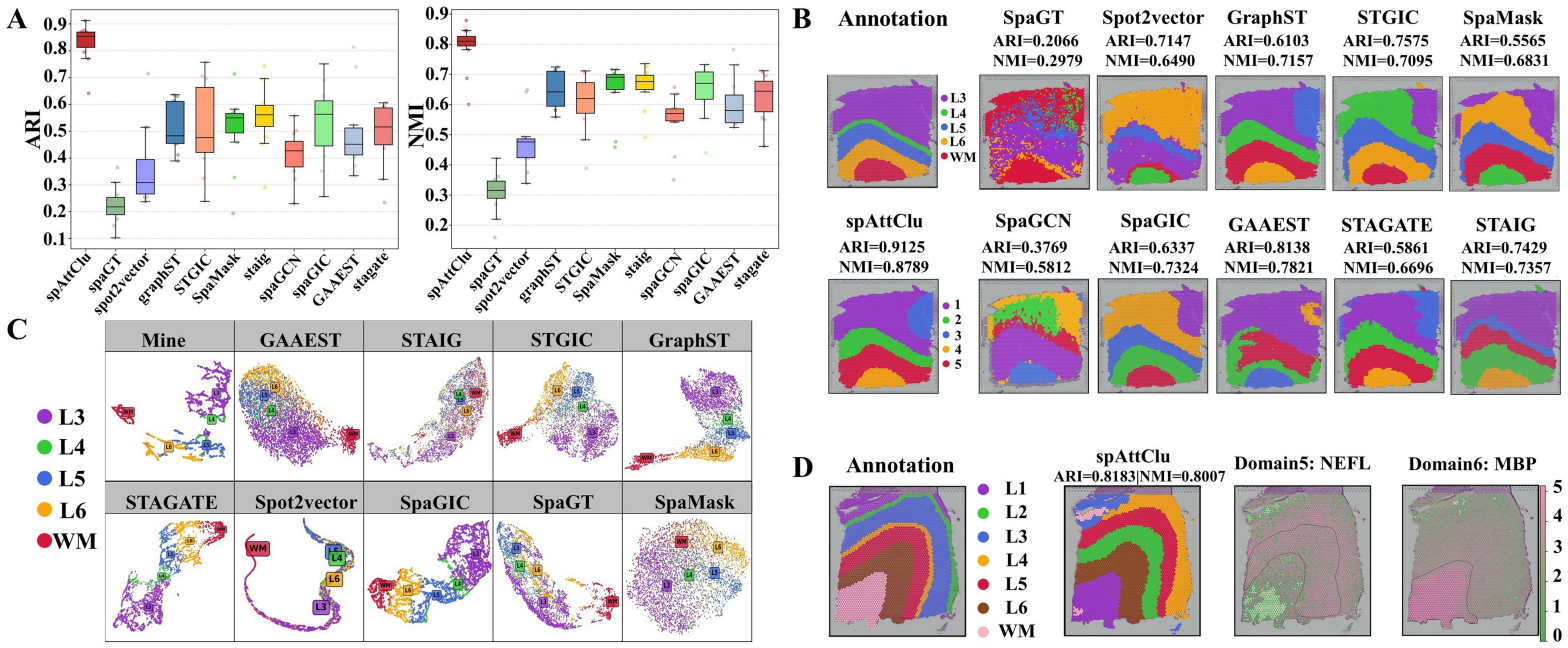
spAttClu improves tissue architecture recognition in human DLPFC. (A) Box plots of ARI (left) and NMI (right) scores for all DLPFC sections. (B) Comparison of spatial domains identified by spAttClu and baseline methods on section #151671. (C) UMAP visualization of embeddings generated by the corresponding methods. (D) Manually annotated layers and spatial expression patterns of SVGs detected by spAttClu for section #151673.

In the overall evaluation across all sections, spAttClu demonstrates superior and stable clustering performance (Fig. 2A, Supplementary S9–S20, available as supplementary data at *Bioinformatics* online), achieving an average ARI of 0.8321 and an average NMI of 0.7950, significantly outperforming all compared methods (the suboptimal method STAIG attained an ARI of only 0.5442, representing a 52% performance improvement by spAttClu). The complete results across all 12 sections, presented in Supplementary S21–S23, available as supplementary data at *Bioinformatics* online, further validate the effectiveness of spAttClu. Taking slice #151671 as an example (Fig. 2B), spAttClu accurately captures the sharp boundaries between layer 6 and white matter (WM) and between layers 5 and 6, while other methods exhibit inter-layer mixing, coarse boundaries, or inaccurate layer thickness. The Wilcoxon signed-rank test *p-*value (Zhang *et al.* 2025) in Table S2 further validate the statistically significant clustering superiority of our model over competing methods. This advantage stems from the model’s spatially weighted graph attention mechanism, which transforms physical distances into adaptive attention priors via a Gaussian kernel, thereby more accurately learning biologically plausible neighborhood relationships and achieving higher, more stable clustering accuracy. UMAP visualization (Fig. 2C) shows that embeddings from spAttClu present a clear continuous trajectory from L1 to L6 and WM, capturing the functional gradient across layers, whereas embeddings from compared methods are loosely structured. Furthermore, clustering reliability was verified by identifying spatially variable genes (SVGs). In slice #151673, NEFL is highly expressed in the L6 domain and MBP is enriched in WM (Min *et al.* 2025) (Fig. 2D), consistent with known cortical marker genes (Wang *et al.* 2024). Furthermore, the GO enrichment analysis results reported in Supplementary S24, available as supplementary data at *Bioinformatics* online provide further support for the effectiveness of our model.

### 3.2 spAttClu exhibits robustness to low gene counts

**Figure 3:**
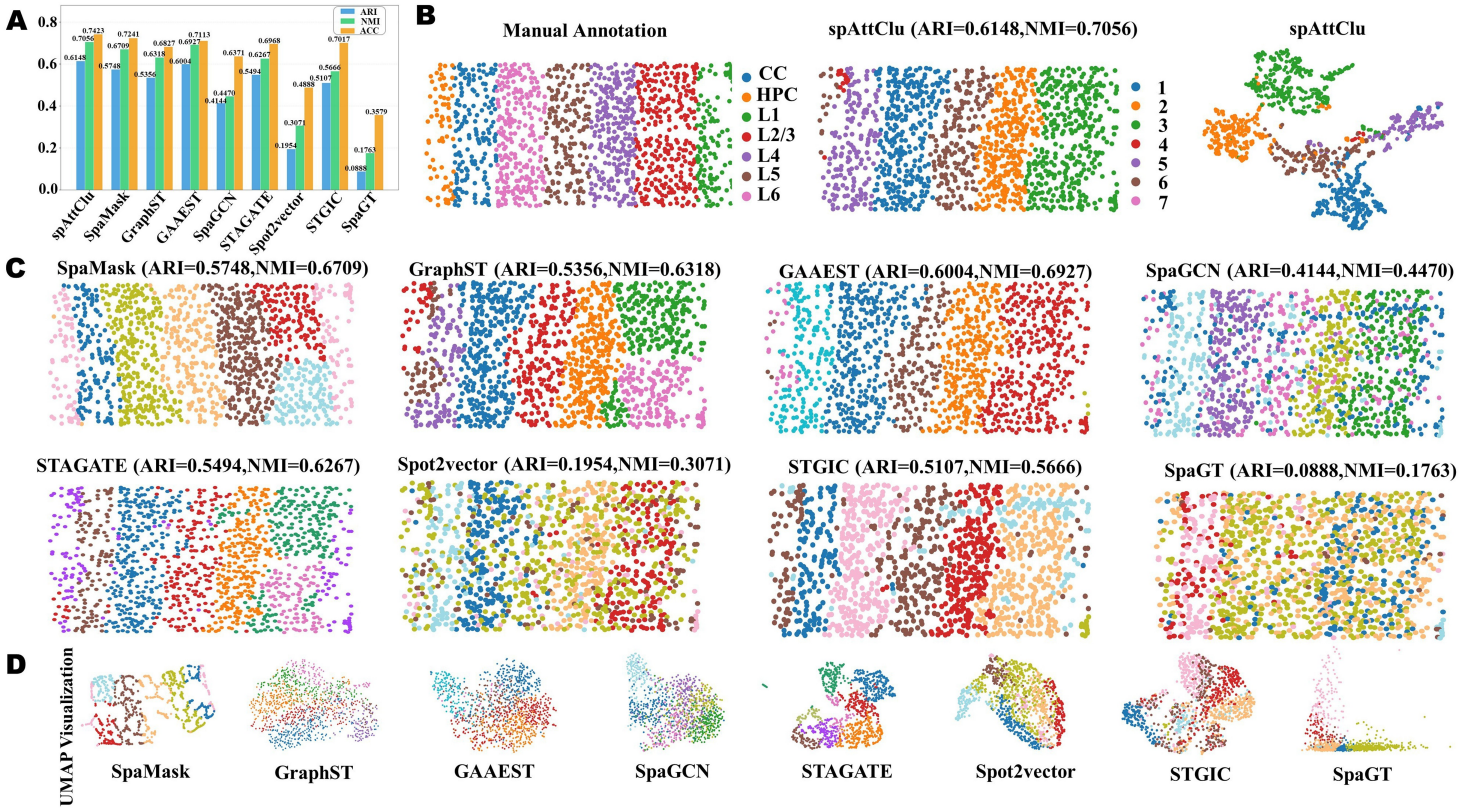
Spatial domain recognition on the STARmap MVC dataset. (A) Bar plot comparing model performance metrics. (B) Ground truth annotation, identified spatial domains by spAttClu, and the corresponding UMAP visualization. (C) Spatial domain recognition results from baseline methods. (D) UMAP visualization of the generated embeddings.

Spatial transcriptomics is often limited by low gene detection rates, which compromises cell type resolution and clustering reliability (Liu *et al.* 2024). To evaluate the robustness of spAttClu under such conditions, this study employs the STARmap mouse visual cortex (MVC) dataset for validation. As shown in Fig. 3A, spAttClu achieves superior ARI (0.6148), NMI (0.7056), and ACC (0.7423) scores compared to all baseline methods, showing an approximately 2.4% improvement over the best baseline GAAEST and more substantial advantages over Spot2vector and SpaGT. This indicates that the model’s dynamic negative sampling strategy in contrastive learning effectively integrates spatial and expression information even with limited genes, maintaining high clustering accuracy.

In spatial domain recognition, the clusters identified by spAttClu are closely aligned with the cortical laminar structure (Fig. 3B), with significantly clearer boundaries than baseline methods. For instance, GraphST shows confusion between L1 and L2/3 layers, while SpaGT produces disordered patchy distributions. By enforcing embedding similarity among neighbors via a spatial consistency loss, spAttClu effectively avoids cluster fragmentation. UMAP visualization (Fig. 3D) reveals that the embeddings from spAttClu exhibit the clearest separation between cortical layers, whereas baseline methods generally suffer from blurred cluster boundaries or disorganized distributions.

### 3.3 spAttClu enables cross-sample recognition of spatial domains through multi-section integration

Spatial transcriptomics is advancing towards multi-sample systematic comparisons. Multi-section integration can identify shared spatial domains across samples, but technical batch effects often confound biological variation. To address this, this study evaluates spAttClu against five advanced methods in both vertical and horizontal integration scenarios to validate its performance on multi-section data. See Supplementary S25, available as supplementary data at *Bioinformatics* online for detailed multi-slice integration method.

**Figure 4:**
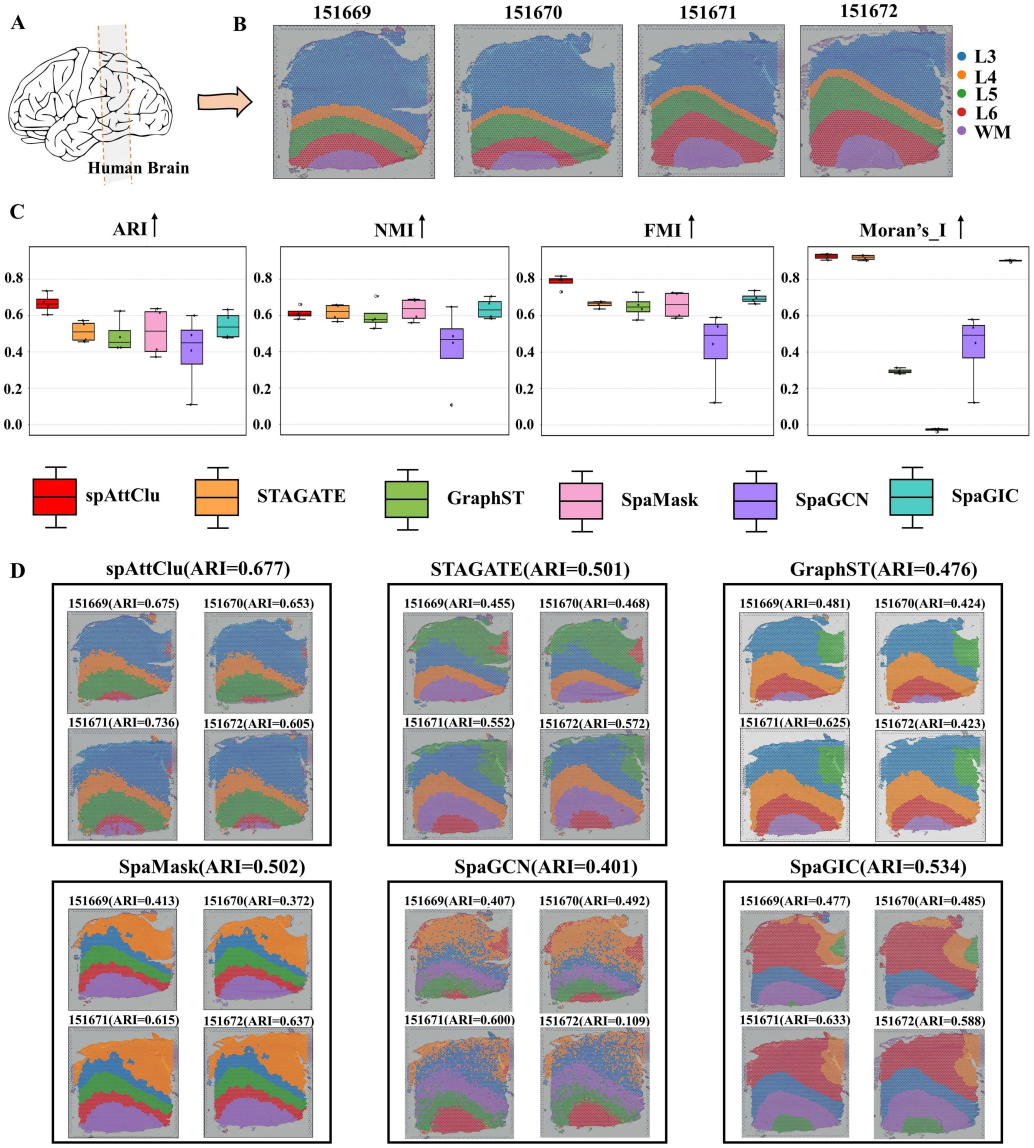
spAttClu corrects batch effects in consecutive DLPFC sections. (A) Schematic of open-source brain regions (adapted from Openclipart). (B) Gold standard annotations of laminar structures for consecutive sections (151 669–151 672). (C) Box plots comparing model performance. (D) Spatial domain recognition results from multiple methods.

In vertical integration using consecutive human DLPFC sections (Fig. 4B), spAttClu achieves section-to-section embedding alignment via its Gaussian kernel-based, spatially weighted attention module, yielding an average ARI of 0.677 and FMI of 0.785 (Fig. 4C). This performance is superior to STAGATE (0.663) and GraphST (0.650). Its average spatial continuity metric (Moran’s I) is 0.925, higher than SpaGCN (0.422). The Moran’s I for spaMask approaches zero, likely because its feature reconstruction and edge prediction losses focus solely on local neighborhood similarity rather than global continuity, resulting in clusters lacking spatial autocorrelation. Visualizations confirm that the cortical structures recognized by spAttClu align closely with the annotations, whereas baseline models show confusion in layers L3/L4 (Fig. 4D).

**Figure 5:**
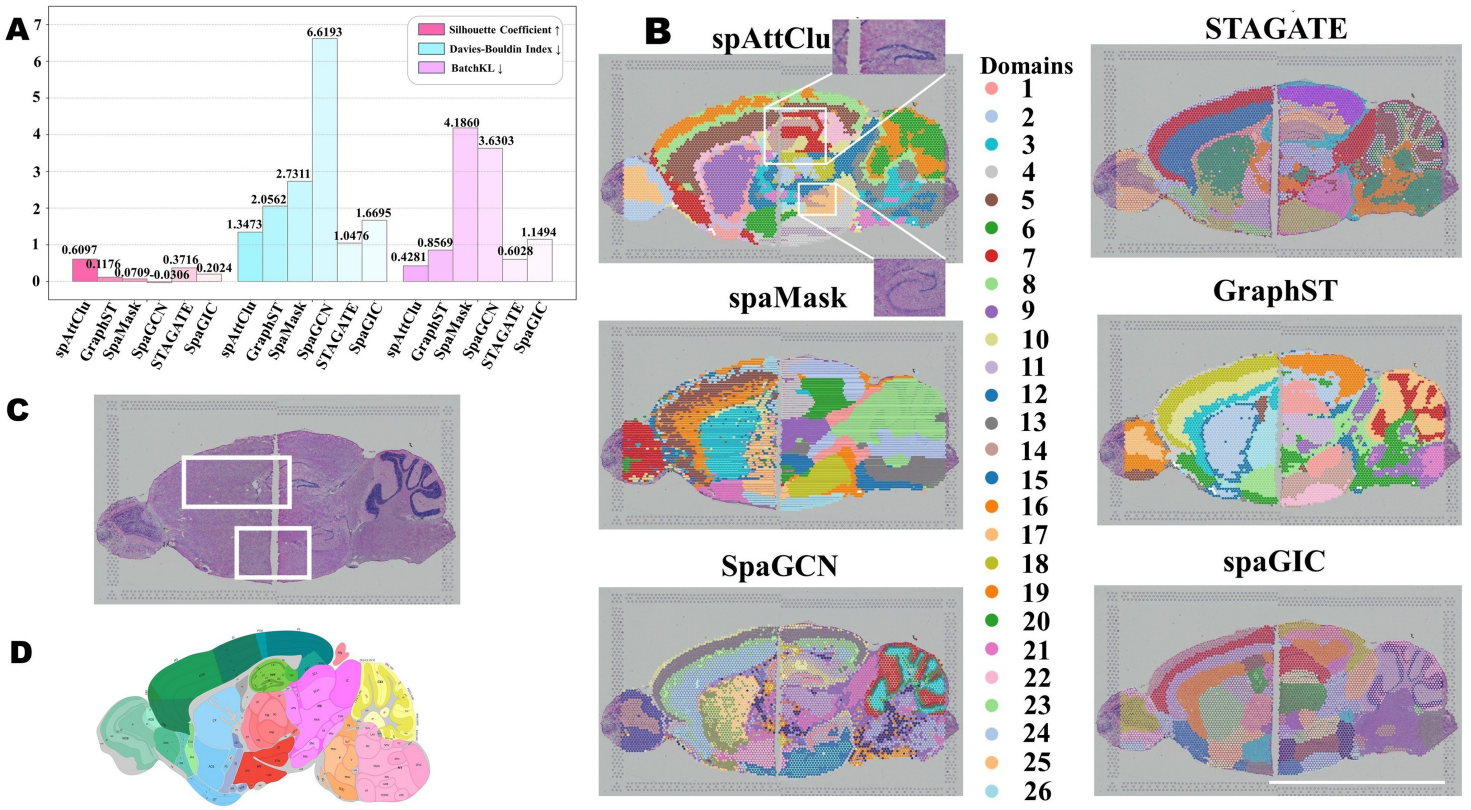
Horizontal integration by spAttClu. (A) Bar plot comparing integration performance metrics. (B) Schematic comparison of hippocampal region identification. (C) H&E-stained image of mouse brain tissue. (D) Image of reference brain atlas annotations.

For horizontal integration using mouse brain anterior/posterior sections, spAttClu first achieves spatial alignment via H&E feature-based hierarchical registration and coordinate mapping (Chen *et al.* 2021), then integrates graph attention to learn cross-section representations. Results show spAttClu achieves the highest Silhouette Coefficient (0.610) and the lowest Davies-Bouldin Index (1.347) (Fig. 5A). It also has the lowest BatchKL score (0.428), indicating effective batch correction. The recognized anatomical structures further validate the method’s biological plausibility (Fig. 5B). While clusters from STAGATE, SpaGIC, GraphST, and spAttClu generally align well with known anatomy (Fig. 5D), SpaGCN yields fragmented clusters misaligned with tissue edges. STAGATE and SpaGIC fail to identify the dorsal and ventral angles of the hippocampal region (Fig. 5C), which spAttClu successfully recognizes.

In conclusion, evaluations in both vertical and horizontal integration scenarios confirm the effectiveness of spAttClu for multi-section data integration.

### 3.4 Robust cross-platform recognition of spatial domains achieved with spAttClu

Spatial transcriptomics platforms (e.g., Stereo-seq, osmFISH, MERFISH) vary significantly in spatial resolution and gene coverage. Such heterogeneity often degrades clustering performance in cross-platform applications. Evaluating a model’s robustness in recognizing spatial domains across platforms is therefore crucial for validating its generalization capability. This study systematically assesses spAttClu’s performance on multiple SRT platform datasets.

**Figure 6:**
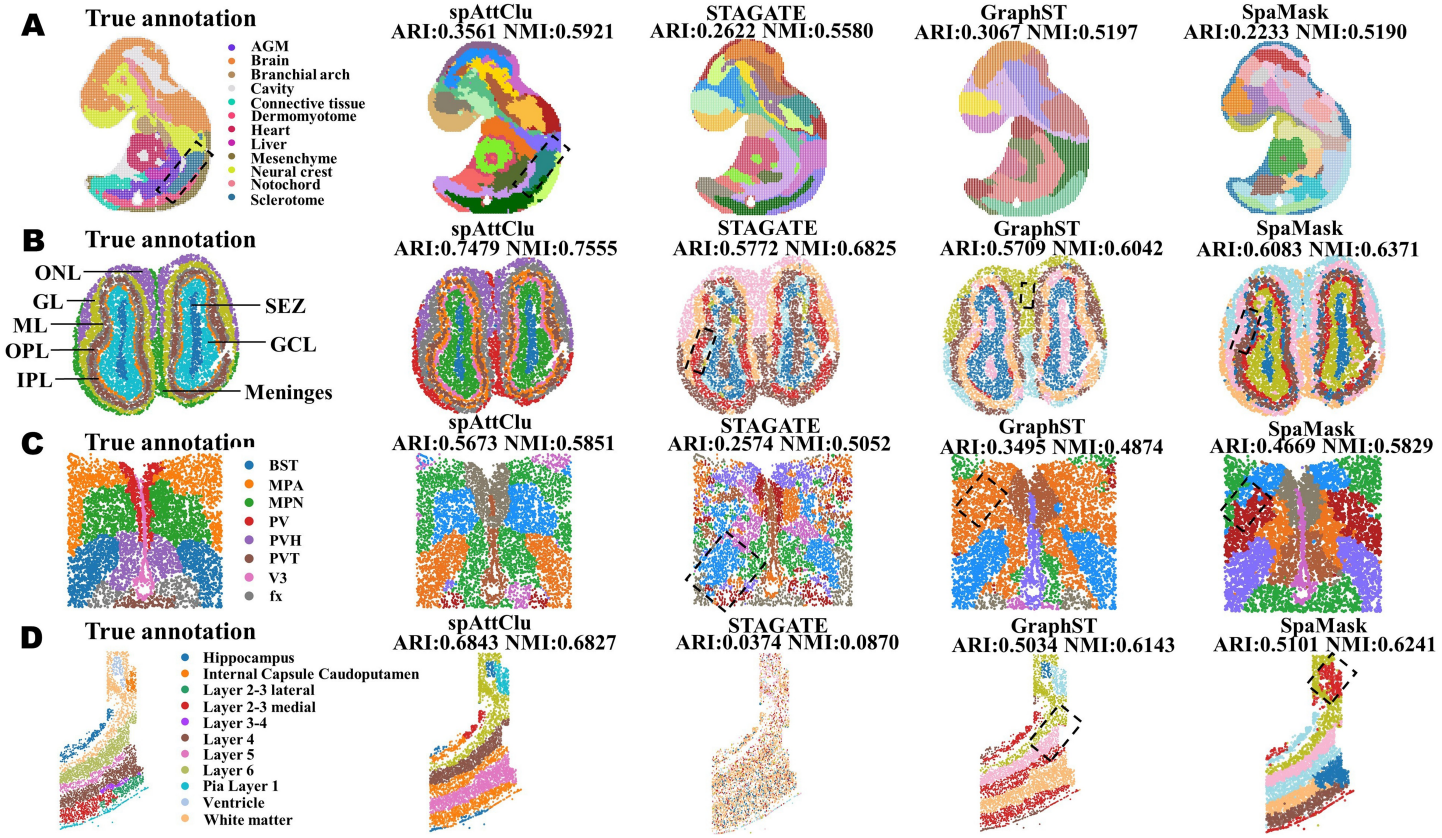
Spatial domain recognition results on cross-platform SRT data. (A) Mouse embryo from the Stereo-seq platform. (B) Mouse olfactory bulb from the Stereo-seq platform. (C) Mouse hypothalamic preoptic area (Bregma −0.09 mm) from the MERFISH platform. (D) Mouse cortex data from the osmFISH platform.

First, spAttClu was applied to high-resolution data from the Stereo-seq platform, including mouse embryo (9.5E) and mouse olfactory bulb datasets. For the mouse embryo data (Fig. 6A), spAttClu identified the complete sclerotome region (ARI = 0.3561, NMI = 0.5921), which was not fully captured by other methods. This success is attributed to the model’s data preprocessing module, which adaptively constructs a k-nearest neighbor graph suited to the dense point distribution characteristic of high-resolution data. In the mouse olfactory bulb data (Fig. 6B), the regions identified by spAttClu closely matched the anatomical annotations, whereas GraphST showed intermixing, and STAGATE and SpaMask failed to distinguish the ML and OPL layers. Quantitative analysis confirmed spAttClu’s superior performance, achieving the highest ARI (0.7479) and NMI (0.7555). This precise recognition of complex layered structures stems from the model’s spatially weighted graph attention mechanism, which incorporates physical distance via a Gaussian kernel to enhance modeling of biological similarity among neighboring spots.

Testing on a MERFISH platform dataset of the mouse hypothalamic preoptic area (Bregma -0.09 mm) further validated model adaptability. This dataset contains eight spatial domains, and spAttClu employed a distance threshold for graph construction. Results (Fig. 6C) show that spAttClu (ARI = 0.5673, NMI = 0.5851) successfully distinguished adjacent brain regions like MPA and MPN, while GraphST and SpaMask erroneously merged them, and STAGATE produced distorted thickness in the BST region. The model’s highest ACC (0.7142) on this dataset indicates that its distance-weighted mechanism can adapt to complex brain region partitioning tasks at varying scales.

To examine performance on non-gridded data, spAttClu was applied to osmFISH platform data from the mouse somatosensory cortex. With only 33 genes, preprocessing involved no Highly Variable Genes (HVG) selection or dimensionality reduction; normalized expression was used directly as input with distance-threshold-based graph construction. Under these conditions, spAttClu achieved excellent clustering performance (ARI = 0.6843) (Fig. 6D), representing a 34.15% improvement over the suboptimal method, SpaMask (ARI = 0.5101). Visualizations showed that STAGATE produced chaotic clusters, SpaMask failed to separate the Ventricle and Internal Capsule Caudoputamen, and GraphST showed abnormal thickness in layer 6. This performance gain is linked to the model’s spatially weighted attention mechanism, which mitigates information sparsity from low gene counts and helps capture subtle expression patterns across cortical layers.

This systematic cross-platform evaluation demonstrates that spAttClu, through its platform-adaptive graph construction and encoder strategy in preprocessing, combined with its spatially weighted attention mechanism, delivers robust clustering performance and generalization capability across data with varying resolutions, gene numbers, and spatial structures.

### 3.5 spAttClu supports the analysis of large-scale datasets

**Figure 7:**
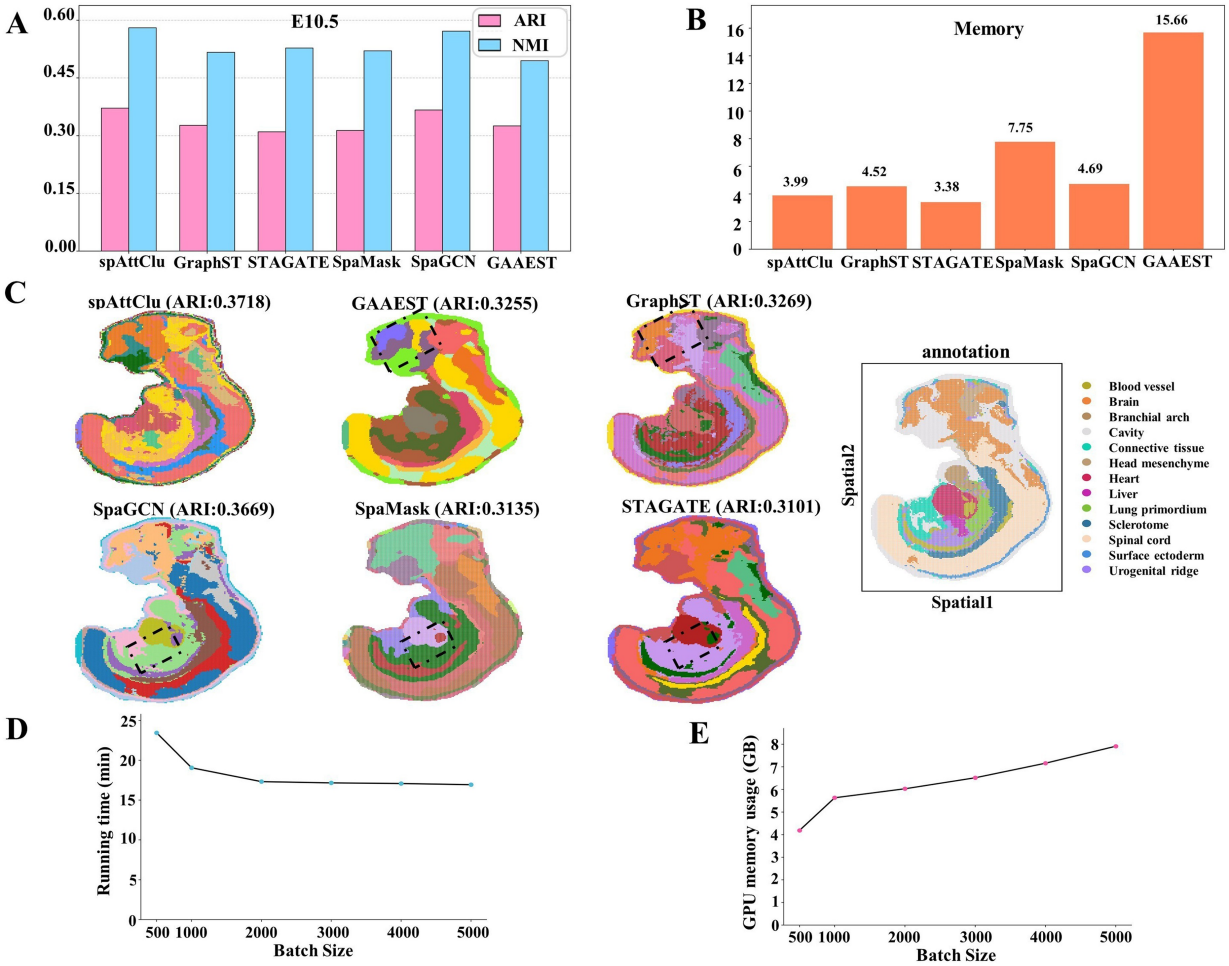
Model performance analysis on the large-scale dataset. (A) Clustering performance of multiple methods on the E10.5 dataset. (B) Memory usage of multiple methods on the E10.5 dataset. (C) Spatial domain visualizations by multiple methods on the E10.5 dataset. (D) Impact of batch size on runtime. (E) Impact of batch size on memory usage.

The advancement of spatial transcriptomics technologies has expanded data scales to tens or hundreds of thousands of spatial units, imposing higher demands on the computational efficiency and memory management of clustering algorithms. To evaluate spAttClu’s performance on large-scale data, this study employs large-scale mouse embryo datasets (E10.5 and E14.5) generated by Stereo-seq for testing. On the E10.5 dataset, spAttClu achieved the best ARI (0.3718) and NMI (0.5806) (Fig. 7A), while its memory consumption (3.87 GB) was significantly lower than that of baseline methods (Fig. 7B). Visualization results show that spAttClu more accurately and clearly identifies brain regions and delineates boundaries between adjacent tissues like the liver and heart compared to other methods (Fig. 7C).

To optimize large-scale data processing efficiency, we systematically evaluated batch size impact on performance using the E14.5 dataset. A batch size of 2000 provides a good balance between efficiency and memory usage (Fig. 7D and E). Additionally, Supplementary S26, available as supplementary data at *Bioinformatics* online shows that, compared to baseline models, our model spAttClu requires the shortest runtime. To validate scalability on larger data, we tested a 640 000-spot simulated dataset. Supplementary S27, available as supplementary data at *Bioinformatics* online shows our model runs effectively on large-scale datasets.

### 3.6 Ablation studies

To validate the effectiveness of each core module in spAttClu, systematic ablation studies were conducted on all 12 sections of the DLPFC dataset. Two key variants were constructed: (1) Ablation-SpatialAttention, which removes the spatially weighted Gaussian kernel attention module, reverting to a vanilla GAT attention mechanism based solely on node feature similarity and thus eliminating spatial prior constraints; and (2) Ablation-Contrastive, which eliminates the contrastive learning and mutual information maximization module, using a pure autoencoder reconstruction loss without instance discrimination or mutual information constraints, thereby hindering the learning of noise-robust, discriminative representations.

Results in Table 2 show that removing the contrastive learning module severely degrades clustering performance, with the average ARI plummeting from 0.83 to 0.31. This module, through dynamic negative sampling and dual-contrastive loss, effectively distinguishes true data distribution from noise. Its absence weakens representation discriminability, correspondingly reducing the average NMI from 0.78 to 0.46, confirming the crucial role of contrastive learning. Removing the spatial attention module reduces the average ARI from 0.83 to 0.44, with a drop of 0.50 observed for section 151670. This module quantifies physical distance into attention weights via a Gaussian kernel; its removal impairs the model’s ability to capture biological associations among spatially proximate spots. The median NMI post-ablation is 0.58, a decrease of 0.21 from the full model’s 0.79, further indicating the module’s effectiveness in dynamically adjusting neighborhood contributions.

**Table 2:** Ablation study data.

Dataset	ARI			NMI		
	Complete Model	Ablation spatial attention	Ablation contrastive	Complete Model	Ablation spatial attention	Ablation contrastive
DLPFC_151507	0.7707	0.5271	0.2254	0.6872	0.6072	0.4602
DLPFC_151508	0.8693	0.3412	0.2740	0.8453	0.5307	0.4989
DLPFC_151509	0.8552	0.4707	0.5066	0.7984	0.5781	0.5491
DLPFC_151510	0.6419	0.3451	0.2913	0.6011	0.5679	0.4561
DLPFC_151669	0.8705	0.3212	0.1996	0.8110	0.4644	0.3036
DLPFC_151670	0.8524	0.3556	0.1146	0.8107	0.5243	0.2582
DLPFC_151671	0.9125	0.6565	0.4233	0.8789	0.6766	0.4901
DLPFC_151672	0.8760	0.7217	0.5196	0.8453	0.7178	0.5809
DLPFC_151673	0.8183	0.5178	0.2171	0.8007	0.6651	0.4185
DLPFC_151674	0.8411	0.5051	0.4540	0.8087	0.6315	0.5867
DLPFC_151675	0.8629	0.4121	0.2395	0.8197	0.5810	0.4346
DLPFC_151676	0.7955	0.3913	0.3492	0.7820	0.5493	0.5006

Collectively, the ablation results demonstrate that the full spAttClu architecture consistently achieves optimal performance on the DLPFC dataset. This confirms the efficacy of both modules: the spatial attention module ensures precise modeling of local spatial context, while the contrastive learning module enhances representation robustness. Their combined contributions underpin the model’s final clustering performance.

**Figure 8:**
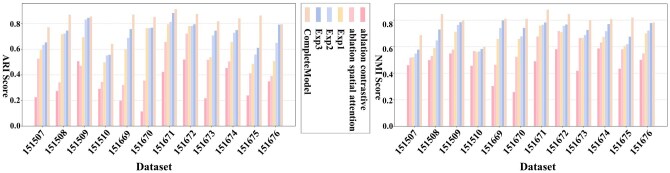
Multi-level ablation experiments.

To further validate the necessity and synergy of the spatially weighted Gaussian attention and contrastive learning modules, multi-level ablation experiments were performed on the DLPFC dataset (Fig. 8). Results show that completely removing either module causes significant performance degradation, confirming their indispensability. In intermediate ablations, replacing the Gaussian prior with a fixed adjacency matrix while retaining DGI (Exp-3) yielded weaker clustering than the full model, indicating the adaptive Gaussian prior more flexibly captures task-driven spatial associations rather than relying on static geometric distance. Conversely, replacing DGI with a simple regularizer while retaining the spatial prior (Exp-2) performed better than Exp-1 but worse than Exp-3, confirming the superiority of the contrastive mechanism in mining discriminative features, which a simple regularizer cannot provide. Removing the spatial continuity loss while keeping DGI (Exp-1) led to performance deterioration, showing that spatial constraints provide necessary inductive bias for contrastive learning; without this guidance, DGI struggles to capture spatially coherent semantic patterns spontaneously. These results demonstrate that the two modules do not operate in isolation but form a tightly synergistic framework: the spatial prior provides structural constraints for contrastive learning, which in turn optimizes feature discriminability and robustness within those constraints. Their synergy drives the model towards more accurate spatial domain clustering in complex spatial transcriptomics data.

## 4 Discussion

The spAttClu framework proposed in this study provides a new analytical tool for the accurate identification of spatial domains in spatial transcriptomic data. The core of our method lies in introducing a spatially distance-weighted graph attention mechanism, which enables the model to dynamically adjust the importance weight of each neighbor based on the local gene expression context of each node while maintaining spatial proximity guidance, thereby achieving adaptive fusion of gene expression and spatial neighborhood information.

To comprehensively evaluate model performance, we systematically tested spAttClu across multiple analytical tasks. In the single-slice clustering task, spAttClu achieved high-precision layer structure identification on all DLPFC slices. In the multi-slice integration task, spAttClu eliminated technical batch effects while preserving biological variation, enabling accurate alignment of spatial domains across samples. Cross-platform validation further confirmed the model’s generalizability, with spAttClu maintaining robust clustering performance on four datasets generated by different technological platforms, including Stereo-seq, osmFISH, and MERFISH. The successful application to a large-scale embryonic dataset extended the applicability of this method in spatial dissection of complex biological systems. Ablation experiments verified the synergistic effects of each core module, supporting the reliability of the methodology in biological research.

Despite its demonstrated advantages, spAttClu has certain limitations. First, its capability to integrate multimodal data is limited. While it can process basic spatial coordinates and gene expression data, its utilization of richer information such as histology images is not yet sufficient. Second, the spatially weighted attention mechanism increases computational complexity, imposing higher demands on resources when processing extremely large datasets (e.g., millions of spots). Furthermore, despite improved stability through regularization and contrastive learning, model performance may still be affected by initialization and exhibit fluctuations on highly heterogeneous datasets, which requires attention in application scenarios emphasizing reproducibility.

To address these, future work will focus on introducing more efficient memory management strategies, such as hierarchical graph processing, to enhance capability for larger-scale data. For multimodal integration, dedicated image feature extraction modules will be developed to achieve deeper fusion of gene expression and tissue morphology. We will also explore more stable graph representation learning mechanisms to further improve the model’s robustness and generalizability across diverse data scenarios.

## Supplementary Material

btag384_Supplementary_Data

## Data Availability

The code supporting this work is accessible at https://github.com/zhangtiancai123/spAttClu.git, with an archived version deposited on Zenodo (https://doi.org/10.5281/zenodo.20350239). The data underlying this work are available within the article.
