# Supplementary material for: spAttClu: a spatial domain clustering model leveraging spatially weighted graph attention and contrastive learning
Source: Bioinformatics. 2026 Jun 13;42(6):btag384. doi: 10.1093/bioinformatics/btag384 (PMC13303289; doi:10.1093/bioinformatics/btag384)
Supplement: btag384_Supplementary_Data [file btag384_supplementary_data.pdf]

Supplementary S1. This study employed multiple spatial transcriptomics datasets to evaluate the performance of spAttClu across various tasks. The detailed information for each dataset is as follows.

Spatial Clustering Task

We utilized the human dorsolateral prefrontal cortex (DLPFC) dataset sequenced using the 10x Visium technology as the benchmark dataset. This dataset comprises 12 tissue sections, with each section containing between 3,460 and 4,789 spots, capturing 33,538 genes. The spatial resolution is 55μm/spot, consistent with the standard resolution of the 10x Visium platform. All sections were manually annotated with 5 to 7 anatomical domains, including cortical layers L1-L6 and white matter (WM), by the original authors. The data were sourced from the publicly available LIBD database (http://research.libd.org/spatialLIBD/).

Multi-Sample Integration Task

Horizontal Integration: We employed a mouse brain dataset sequenced using the 10x Visium technology. This dataset comprises two sections, anterior and posterior, sourced from the 10x Genomics public repository (https://www.10xgenomics.com/resources/datasets/).

Vertical Integration: We utilized 4 consecutive sections (serial numbers: 151669 to 151672) from Donor 3 of the aforementioned human DLPFC dataset. These sections exhibit inherent inter-section batch effects, making them suitable for evaluating batch effect correction capability. Each section was manually annotated with cortical layers (L3-L6) and white matter (WM) domains.

Cross-Platform Spatial Domain Recognition Task

To validate the model's robustness across platforms with different resolutions and gene coverages, we utilized the following four datasets:

1.Stereo-seq Mouse Embryo Dataset (E9.5): This data was used to test performance on high-resolution complex organs. The tissue is an E9.5 intact mouse embryo, comprising 5,913 bins and 23,015 genes. Data were sourced from the CNGB STOmics database (https://db.cngb.org/stomics/mosta/).

2.Stereo-seq Mouse Olfactory Bulb Dataset: This data was used to test the capability for fine partitioning of laminar structures. This dataset, derived from the study by Chen et al., contains one coronal section with approximately 10,000 spots, covering 26,145 genes, and boasts a spatial resolution as high as 220 nm. This dataset has been manually annotated into 8 distinct laminar domains.

3.osmFISH Mouse Somatosensory Cortex Dataset: This data was used to test performance on non-gridded platforms with a low number of genes. The tissue is the mouse somatosensory cortex, exhibiting a 6-layer laminar structure.

4.MERFISH Mouse Hypothalamic Preoptic Area Dataset: This data was used to test performance on high-resolution data with a low number of genes from a specific brain region.

Low Gene Count Robustness Task

We utilized a mouse visual cortex dataset sequenced using STARmap technology. The dataset features a subcellular spatial resolution and comprises 1,207 cells (spots), but captures only 1,020 genes, making it ideal for testing scenarios with low gene coverage. This section was manually annotated with 8 functional domains.

Large-Scale Data Scalability Task

We utilized mouse embryo datasets (E10.5 and E14.5) sequenced using Stereo-seq technology. The E10.5 embryo data comprises 18,408 bins and 25,201 genes; the E14.5 embryo

data, with a significantly larger scale, contains 92,928 bins and 18,566 genes, and was used to test the algorithm's capability for processing large-scale data. The data were sourced from the CNGB STOmics database (https://db.cngb.org/stomics/mosta/). We utilized a simulated dataset comprising 31,493 genes and 640,000 spots, partitioned into 9 domains. This dataset was employed to validate the model's scalability on even larger-scale data.

Supplementary S2. To comprehensively evaluate the performance of the spAttClu model in clustering spatial transcriptomics data, this study employs external evaluation metrics and internal evaluation metrics based on whether the data possess known ground truth spatial domain labels. Furthermore, considering the characteristics of spatial transcriptomics data, spatial specificity metrics are introduced to assess the quality of spatial distribution for the recognized domains. To clearly elucidate the differentiated roles of each metric and support the key rationale for subsequent conclusions, the definition, calculation method, and experimental usage of each metric are described below in turn.

When the ground truth spatial domain labels (e.g., manually annotated tissue layer structures) of a dataset are known, in addition to the ARI and NMI evaluation metrics already presented in the main text, we also adopt the following external metrics to compare the clustering results against the ground truth labels, thereby assessing their partitioning accuracy:

Accuracy (ACC): ACC calculates the proportion of correctly classified samples after optimally mapping the predicted cluster labels to the ground truth labels using the Hungarian algorithm. Its calculation is based on maximizing the matching of the contingency table:

$$ACC = \frac{max_{matching} \sum_i n_{i,mapped(i)}}{n}$$

where $n_{i,j}$ shares the same definition as in the ARI, and mapping refers to the optimal cluster label mapping. The value of ACC ranges between [0, 1], with higher values indicating better accuracy. This metric is primarily used as a supplementary valida (ACC, FMI, SC, DB, Moran's I) (ACC, FMI, SC, DB, Moran's I)tion, such as in evaluating model robustness to low gene counts, where it is jointly used with ARI/NMI to enhance the reliability of the conclusions.(Min, et al., 2025)

Fowlkes-Mallows Index (FMI): For paired samples belonging to the same category, the FMI measures the fitting degree between the clustering results and the ground truth labels by calculating the similarity. Its value range is [0,1], and a value closer to 1 indicates a higher level of consistency.

$$FMI = \frac{TP}{\sqrt{(TP + FP)(TP + FN)}}$$

where TP and FP denote the number of correctly or incorrectly clustered sample pairs within the ground truth labels, respectively. FN represents the number of positive clustering sample pairs that were not correctly identified. This metric is used to evaluate clustering fidelity when vertically integrating DLPFC layers in multi-slice joint analysis.(Gong, et al., 2025)

For datasets lacking known ground truth spatial domain labels, internal metrics based on the intrinsic data structure are employed to evaluate clustering quality, primarily considering the compactness of samples within clusters and the separation between samples from different clusters:

Silhouette Coefficient (SC): The SC comprehensively evaluates the compactness of each sample with its own cluster (a) and its separation from the nearest other cluster (b). Its calculation formula is:

$$s(i) = \frac{b(i) - a(i)}{max\{a(i), b(i)\}}$$

where $a(i)$ is the average distance between sample i and all other samples in the same cluster, and $b(i)$ is the average distance between sample i and all samples in the nearest neighboring cluster. The SC for the entire dataset is the average of $s(i)$ over all samples. The value of SC ranges from [-1, 1]. A higher SC value indicates that points within a cluster are close together and distant from other clusters, suggesting a clear cluster structure; a negative value implies that samples may have been assigned to incorrect clusters. This metric is used to evaluate intra-cluster compactness and inter-cluster separation when horizontally integrating mouse forebrain and hindbrain in multi-slice joint analysis.(Yang, et al., 2025)

Davies-Bouldin Index (DB): The DB index is calculated as the average ratio of the sum of within-cluster dispersions for any two clusters to the distance between their centers. Its calculation formula is:

$$DB = \frac{1}{K} \sum_{i=1, j \neq i}^{K} max\left(\frac{\overline{d_i} + \overline{d_j}}{d(c_i, c_j)}\right)$$

where K is the number of clusters, $\overline{d_i}$ is the average distance from all samples in cluster i to its centroid c_i, and $d(c_i, c_j)$ is the distance between centroids c_i and c_j. The DB index is a non-negative value, where lower values indicate tighter clusters, greater separation between clusters, and better clustering performance. This metric is jointly used with SC in the analysis of horizontal integration of mouse forebrain and hindbrain, evaluating clustering quality from a complementary perspective of inter-cluster separation.(Yang, et al., 2025)

Batch KL Divergence (BatchKL): BatchKL assesses the degree of batch effect mixing within the clustering results. It calculates the KL divergence between the batch distribution within each cluster and the uniform distribution (representing ideal mixing), and then takes the average:

$$BatchKL = \frac{1}{K}\sum_{k=1}^{K} KL(P_{bat\quad |k}||U_{batch})$$

where $P_{batc\ |k}$ is the batch distribution within cluster k, and U_{batc} is the uniform distribution. BatchKL is a non-negative value, where lower values indicate more uniform mixing of different batches within the clustering results, signifying better batch correction performance. This metric is specifically used to quantify the degree of residual batch effects when horizontally integrating mouse forebrain and hindbrain in multi-slice joint analysis.(Yang, et al., 2025)

To further validate the discreteness and continuity of the identified spatial domains in physical space, the following spatial metrics are introduced:

Moran's I: Moran's I measures the spatial autocorrelation of gene expression or cluster labels in spatial transcriptomics data, i.e., the degree to which similar values cluster in space. Its calculation formula is:

$$I = \frac{N \sum_i \sum_j w_{ij} (x_i - \bar{x})(x_j - \bar{x})}{W \sum_i (x_i - \bar{x})^2}$$

where N is the number of samples, x_i and x_j are the observed values at locations i and j respectively, $\bar{x}$ is the mean value, w_{ij} is an element of the spatial weight matrix, and $W = \sum_i \sum_j w_{ij}$ is the sum of all weights. The value of Moran's I approximately ranges from [-1, 1]. A positive value indicates positive spatial autocorrelation, a negative value indicates negative spatial autocorrelation, and 0 indicates spatial randomness. In the context of clustering to recognize spatial domains, a higher positive Moran's I value indicates that the identified spatial domains exhibit continuity in physical space. This metric is used to evaluate spatial domain continuity when vertically integrating DLPFC layers in multi-slice joint analysis.(Wang, et al., 2025)

Through this multi-dimensional and comprehensive metric system, this study can comprehensively and objectively evaluate the performance of the spAttClu model and other

comparative algorithms in the task of clustering spatial transcriptomics data.

Supplementary S3. Hyperparameter settings of spAttClu and other involved methods

spAttClu **spAttClu** is a spatial domain clustering model based on spatially-weighted graph attention and contrastive learning. In the preprocessing stage, the built-in preprocess function automatically performs sc.pp.highly_variable_genes (default: 3000 highly variable genes), normalization, log transformation, and scaling, following a fixed pipeline. To ensure fairness across different clustering methods and numbers of highly variable genes (HVGs), spAttClu by default selects 3000 HVGs (implemented via the `n_top_genes=3000` parameter of `sc.pp.highly_variable_genes`), thereby avoiding uncertainty introduced by differences in HVG counts. The adjacency graph is constructed by default based on Euclidean distance and nearest neighbors. Specifically, the parameter K for the k-Nearest Neighbors (k-NN) algorithm in this study was set according to the data characteristics and analytical conventions of each spatial transcriptomics platform. The settings were as follows: for data from the 10x Visium, STARmap, osmFISH, and MERFISH platforms, K was set to 3; for standard Stereo-seq platform data, K was set to 4; and for large-scale Stereo-seq datasets (such as the E10.5 and E14.5 mouse embryos), K was adjusted to 3 to accommodate their higher data density and complexity. This parameter configuration aims to achieve an optimal balance of local spatial structure across different platform environments. The distance weight sigma is automatically calculated from the data median and may vary across datasets. The training epochs are set to 600 by default, using the Adam optimizer with a learning rate of 0.001. The default clustering method is mclust, with support for leiden / louvain (where the resolution is searched to match the number of clusters).

Model training is completely decoupled from downstream clustering, learning only a low-dimensional representation; clustering algorithms (e.g., mclust, leiden, or louvain) are called independently after training, and their choice does not inversely affect model parameters, thus causing no model bias due to different clustering methods. Furthermore, to ensure full reproducibility of all results, a fixed random seed (41) was consistently applied to all experiments involving baseline methods, our proposed approach and ablation analyses; meanwhile, a unified fix_seed function was developed to configure deterministic random seeds for Python, NumPy, PyTorch (CPU and GPU), and CuDNN. The above design guarantees comparability and fairness of experimental results.

spaGT **spaGT** is a model that employs EGT for clustering spatial transcriptomics data. We performed spatial domain clustering following the tutorials on the official GitHub repository and the dedicated documentation website (https://spagt-tutorial.readthedocs.io/). Preprocessing involved log-transformation and normalization of the raw data using SCANPY; the top 3000 highly variable genes were selected. Graph parameters included a spatial adjacency graph based on Euclidean distance (n_neighbors=10) and a reconstruction strength (alpha=1.0). For training settings, the default maximum epoch was 200, the early stopping tolerance was 1e-4, and the update interval was 50. Clustering initialization used mclust, and the number of clusters was manually set based on prior knowledge (e.g., 7 for DLPFC_151507). To ensure reproducibility, random seeds were fixed via setup_seed in the code.

spot2vector **spot2vector** is a deep learning model combining graph attention networks and autoencoders to recognize spatial domains by integrating information from spatial and expression graphs. We conducted clustering analysis following the tutorial provided in the official GitHub

repository. Its preprocessing pipeline includes PCA dimensionality reduction (default n_comps=10), with the spatial graph constructed using radius cutoff (radius_cutoff=150) and the expression graph constructed using KNN cutoff (neighbors_cutoff=4), both generating unweighted adjacency matrices. The default clustering method is mclust, with random seeds fixed to ensure reproducible results.

graphST **graphST** is a graph self-supervised contrastive learning model that integrates graph neural networks with self-supervised contrastive learning to recognize spatial domains. We performed clustering analysis according to the tutorial in the official GitHub repository. Preprocessing involved log transformation, library size normalization, and scaling; the top 3000 HVGs were selected. The graph parameter k was set to 3 to construct an undirected neighborhood graph. Clustering defaulted to mclust. For multi-layer training, we followed their tutorial on vertical SRT integration, using PASTE2-aligned anndata files as input.

STGIC **STGIC** is a deep learning model that combines Adaptive Graph Convolution (AGC) and a Dilated Convolution Framework (DCF) to recognize spatial domains by utilizing graph and image convolutions. We followed the clear steps outlined in the original paper and the tutorial from the official GitHub repository. Preprocessing involved filtering low-expression genes, normalization, followed by natural log transformation; the top 3000 HVGs were selected. Graph parameters utilized a Gaussian kernel to construct the adjacency matrix. Clustering first employed AGC spectral clustering + K-means pre-clustering, with the DCF output determining the final number of clusters. Random seeds were fixed prior to training, ensuring experimental reproducibility.

SpaMask **SpaMask** is a dual-masked graph autoencoder model that combines Masked Graph

Autoencoder (MGAE) and Masked Graph Contrastive Learning (MGCL) to recognize spatial domains. We operated according to the tutorial from the official GitHub repository. The default setting selected 2000 HVGs; graph parameters used KNN to construct the adjacency matrix. The default clustering setup employed the K-means method, with the number of clusters pre-specified based on the dataset (e.g., set to 7 for DLPFC_151507). The random seed was fixed at 2024, ensuring experimental reproducibility.

staig **staig** is a deep learning model based on graph neural networks that utilizes multi-view contrastive learning and graph convolutional networks to recognize spatial domains. We performed spatial domain clustering following the tutorial in the official GitHub repository. Preprocessing involved SCANPY log transformation, normalization, and scaling; 3000 HVGs were selected by default, with the intersection of 5000 genes taken for multi-slice data. Graph parameters used KNN (k=5) to construct the adjacency matrix. The default clustering method was mclust.

spaGCN **spaGCN** is a deep learning model based on Graph Convolutional Networks (GCN). We performed spatial domain clustering following the tutorial in the official GitHub repository. The preprocessing pipeline included standardized gene filtering and normalization steps, with the first 50 principal components extracted via PCA serving as input features for the GCN. Initialization used the Louvain method; when the number of clusters was known, the resolution was set so that the resulting number of clusters matched the known number of spatial domains. This ensured that the initial cluster centers aligned closely with the actual tissue structure before proceeding to the iterative optimization step.

spaGIC **spaGIC** is a graph-based self-supervised contrastive learning framework that

integrates graph convolutional networks with self-supervised contrastive learning to recognize spatial domains by utilizing gene expression data and spatial information. We performed spatial domain clustering following the code tutorial provided in the official GitHub repository. Highly variable gene selection consistently employed the Seurat V3 method to select 3000 genes; clustering defaulted to Mclust.

GAAEST **GAAEST** is a spatial transcriptomics analysis model based on graph attention networks and contrastive learning, which employs graph neural networks and a contrastive learning strategy to recognize spatial domains. We performed spatial domain clustering according to the tutorial in the official GitHub repository. HVGs were consistently selected as the top 3000; graph parameters were constructed via KNN (k=3), with the adjacency matrix regularized; the training budget was set to 600 iterations, and clustering defaulted to Mclust.

stagate **stagate** is a deep learning framework based on graph attention autoencoders that integrates spatial information with gene expression data to recognize spatial domains. We performed spatial domain clustering following the tutorial in the official GitHub repository. HVGs were consistently selected as the top 3000; the graph parameter was SNN (with 6 neighbors set for 10x Visium), and the training budget was fixed at 500 epochs. The clustering setup adopted the default Mclust method.

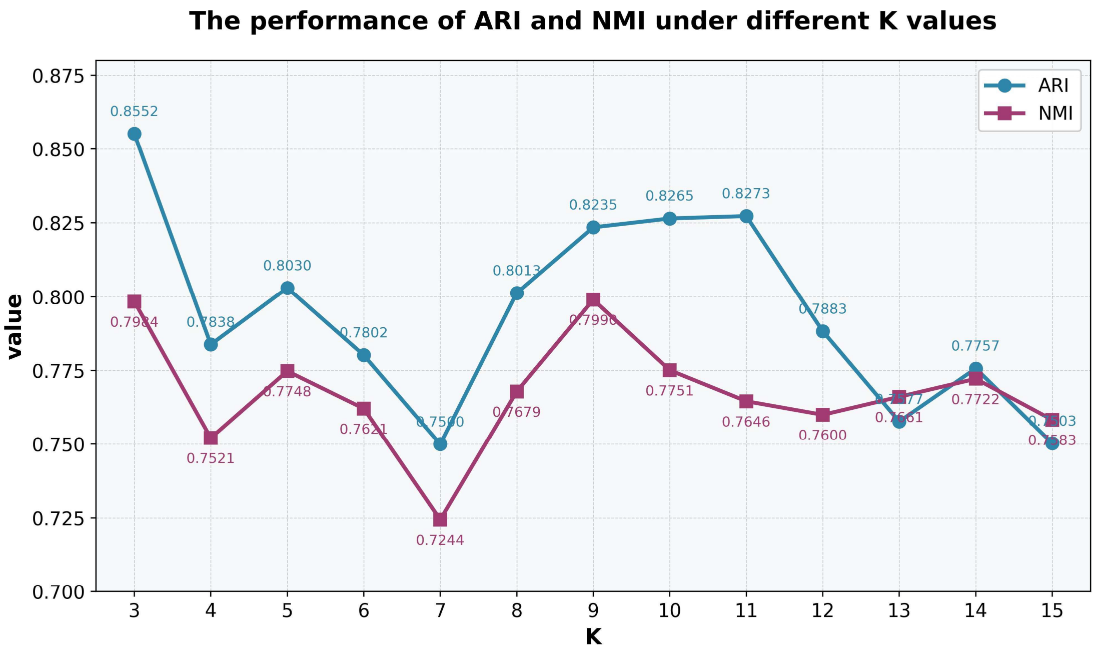

The ARI and NMI performance of our model on the DLPFC 151509 dataset under varying K-values

Supplementary S4. The spatial graph is represented by an adjacency matrix $A \in \{0,1\}^{N\times N}$, where A_{ij}= 1 if and only if spots i and j are spatial neighbors. To further incorporate spatial priors, the resulting weight matrix W serves as the prior weight input for the subsequent graph attention network. This module introduces a Gaussian kernel function to weight spatial distances. Furthermore, to support contrastive learning, this module generates augmented features X_a by randomly permuting the node feature matrix $X \in R^{N\times D}$, and constructs a label matrix $Y \in \{0,1\}^{N\times 2}$ to distinguish positive and negative sample pairs.

To efficiently process large-scale spatial transcriptomics data, the proposed model draws upon the concept of GraphSAGE for handling large-scale graph neural networks, incorporating sparse tensor operations, batching strategies, and dynamic memory optimization techniques. Specifically, during spatial loss computation, loss is calculated only for node pairs corresponding

to non-zero entries in the adjacency matrix, thereby avoiding full-matrix operations. By setting an adjustable batch size, the computation of large-scale edge sets is decomposed into multiple mini-batches to reduce GPU memory consumption. Simultaneously, the model employs a selective attention mechanism that computes attention weights solely between adjacent nodes, thereby lowering computational complexity. Furthermore, memory usage is further optimized by compressing the distance weight matrix via a Gaussian kernel function and storing intermediate results in sparse matrix formats. These strategies ensure the computational efficiency and scalability of the model when processing tens of thousands of spatial nodes. In multi-section integration, the construction of the spatial graph varies according to the integration type: for vertical integration (consecutive sections), a global K-nearest neighbor graph is constructed after aligning the sections into a unified coordinate system via ICP registration; for horizontal integration (non-consecutive sections), within-section and cross-section K-nearest neighbor connections are explicitly established to form a cross-section joint graph, which is combined with distance-weighted attention and spatial regularization loss to preserve spatial continuity.

Supplementary S5. The ARI and NMI performance under different σ values on the DLPFC151509 dataset.

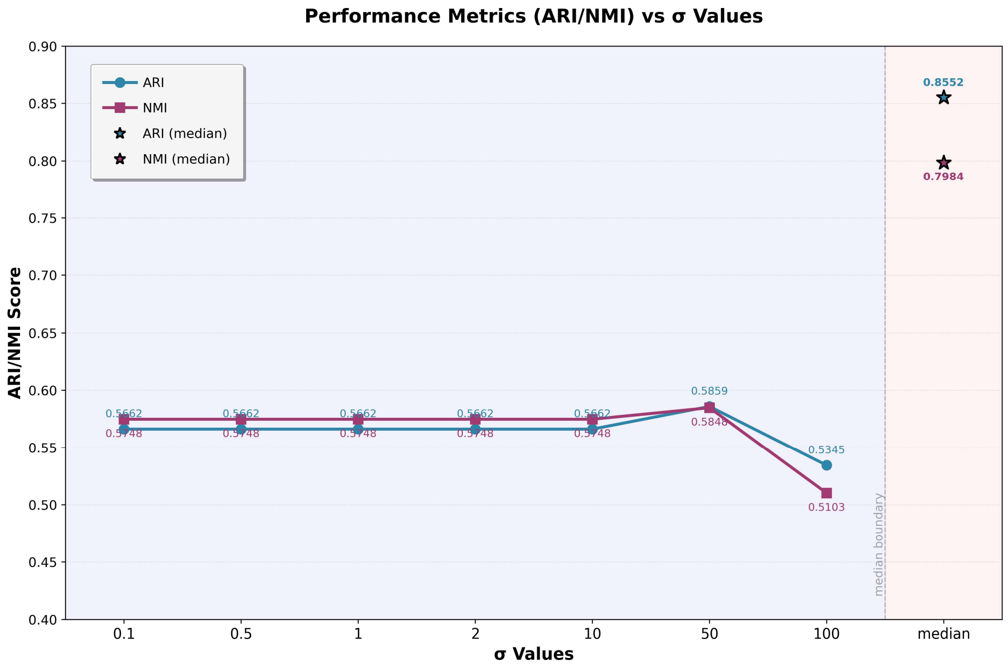

Supplementary S6 To verify the effectiveness of the adopted Gaussian kernel spatial weighting strategy, we performed quantitative evaluation on the STARmap mouse visual cortex dataset. This strategy constructs Gaussian kernel weights via an adaptive bandwidth σ (median of non-zero entries in the distance matrix) and introduces spatial proximity as a differentiable prior into attention coefficients, achieving a synergistic trade-off between gene expression and physical distance. Experimental results show that Gaussian kernel weighting (W0) significantly outperforms no spatial weighting (W2: ARI = 0.5384) and uniform weighting (W1: ARI = 0.5997) in clustering accuracy, and achieves higher performance than inverse distance weighting (W3: ARI = 0.5997) (W0: ARI = 0.6148, NMI = 0.7056). This advantage stems from the smooth decay property of the Gaussian kernel, which adaptively matches the continuous spatial resolution of the tissue, enhancing local neighborhood information while effectively suppressing long-distance noise, thereby learning a more biologically meaningful low-dimensional embedding. This confirms the rationality and superiority of our strategy in integrating spatial context.

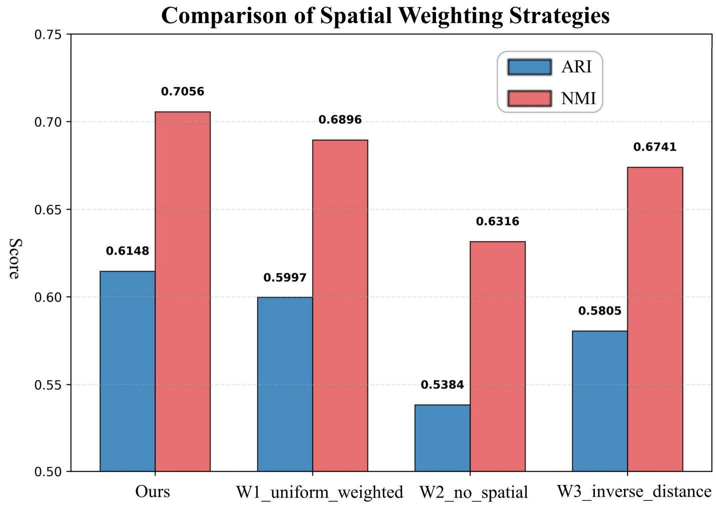

Supplementary S7. As shown in the figure, the systematic sensitivity analysis of the three parameters α, β, and γ indicates that global optimal performance is achieved at α = 10, β = 1, γ = 0.1 (our original selection). Larger α yields better performance and maintains a relative advantage across different combinations of β and γ, suggesting that the model has a positive sensitivity to α within this range. β ∈ {0.1, 1} is a stable interval, implying that the contrastive loss weight should not be excessively large. A small γ (0.1) facilitates the effective functioning of spatial regularization, whereas an overly large γ leads to performance degradation. Therefore, the spAttClu model exhibits good robustness to α and γ within reasonable ranges (α ≥ 1, γ ≤ 1), while β should be kept from taking overly large values. The original parameter selection already resides in the optimal and stable parameter space.

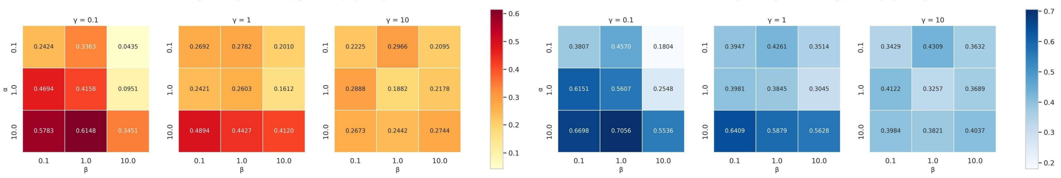

Supplementary S8. The spAttClu model was implemented in Python 3.8.20 and PyTorch 2.0.1+cu117, with core dependencies including bioinformatics analysis libraries such as scanpy and scikit-learn. The model adopts a spatially-weighted graph attention encoder-decoder architecture. Key parameters include: encoder input/hidden/output dimensions of 3000/256/64 respectively, the

Adam optimizer was employed with a learning rate set to 0.001, and the training epochs were 600. Experiments were conducted on a server equipped with an NVIDIA A100 GPU (80GB VRAM).

Supplementary S9. This section presents a visual comparison of spatial domain clustering results on the DLPFC151507 slice from the DLPFC dataset. Manual annotations are shown alongside outputs from 10 methods. Through intuitive visual comparison, it can be observed that our model generates smoother boundaries for the Layer 3 region without boundary fragmentation, which verifies its effectiveness in spatial domain identification.

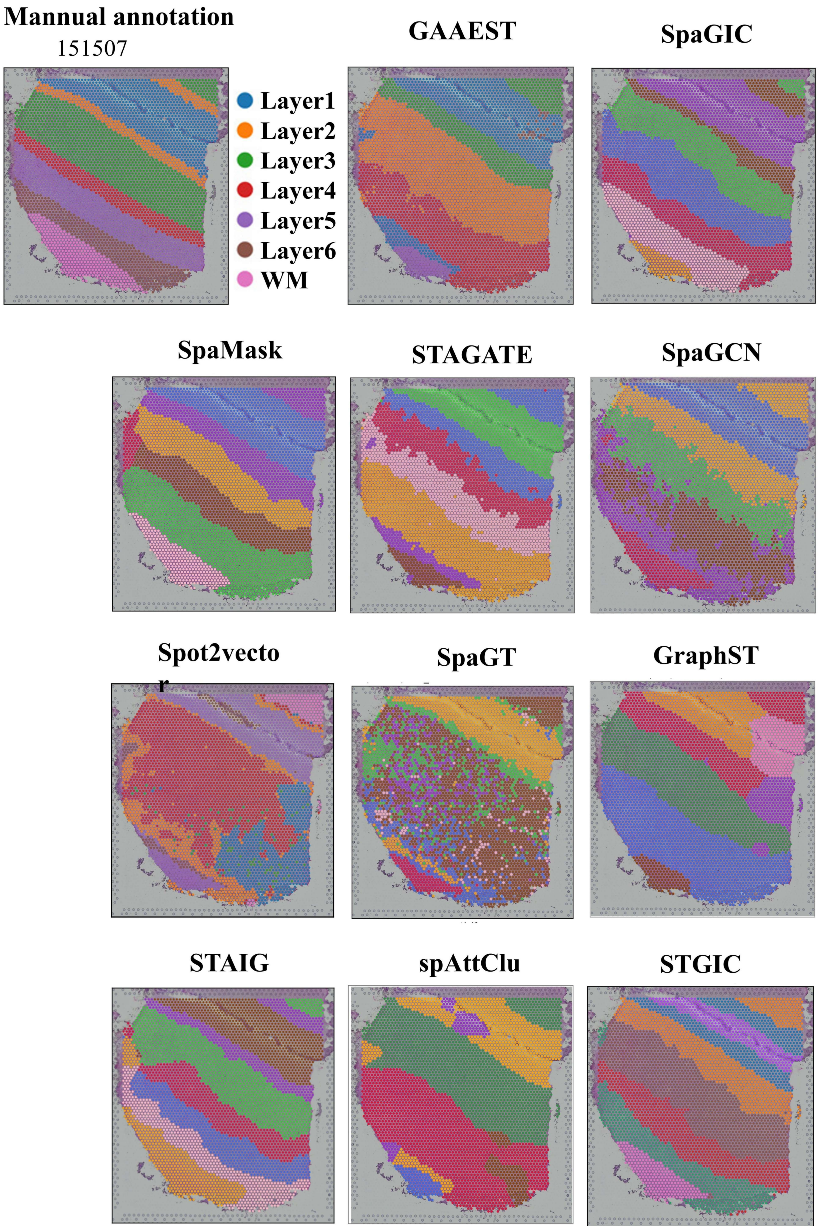

Supplementary S10. This section presents spatial domain clustering results on the DLPFC151508 slice of the DLPFC dataset. The proposed spAttClu model exhibits favorable performance in the spatial partitioning of the white matter (WM) layer along the thickness dimension, which indicates its capacity to accurately delineate WM boundaries.

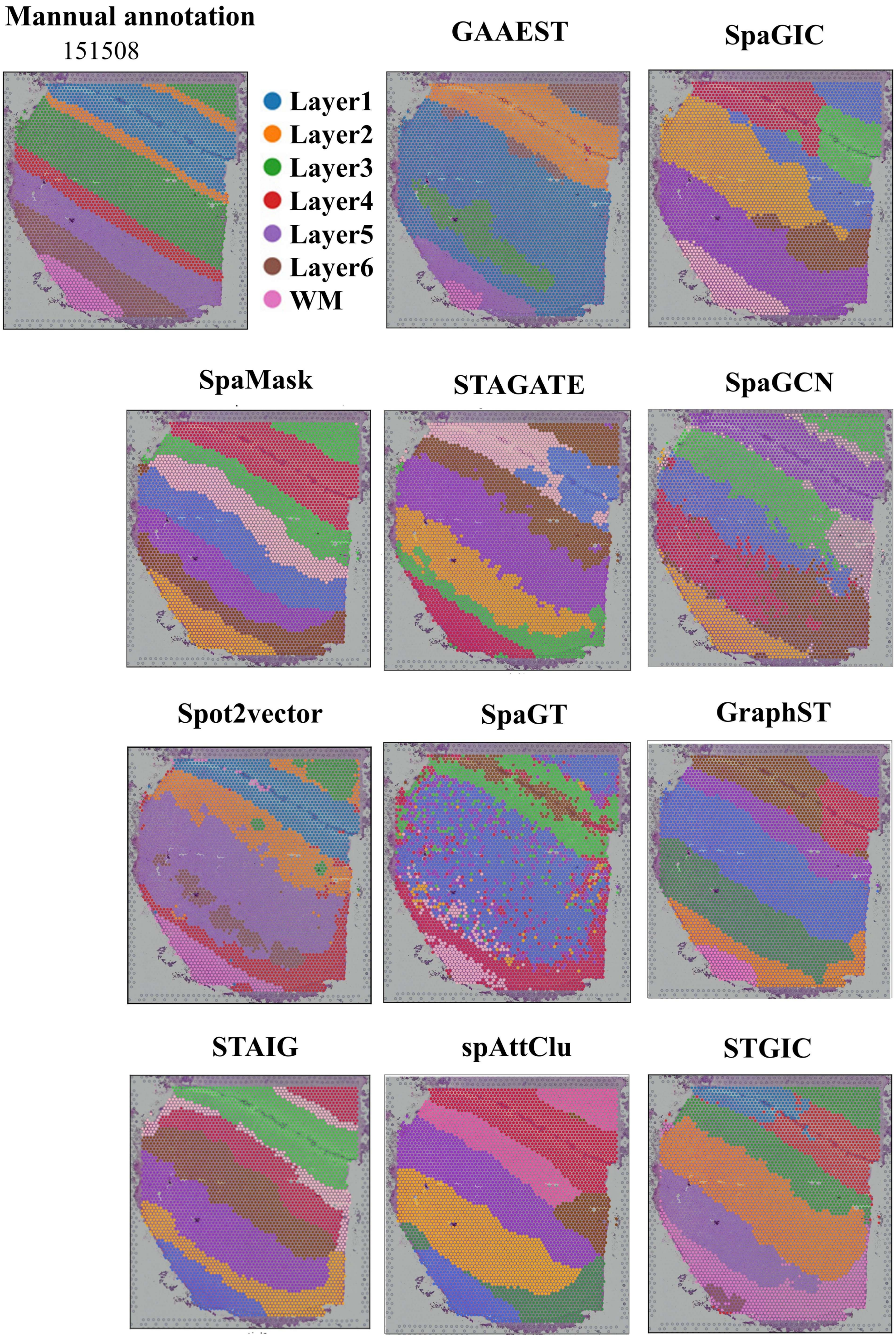

Supplementary S11. This section presents spatial domain clustering results on the DLPFC151509 slice of the DLPFC dataset. The proposed spAttClu model shows reasonable performance in spatial partitioning of the Layer 3 region, achieving a relatively clean delineation with few spurious small patches and a plausible estimation of layer thickness.

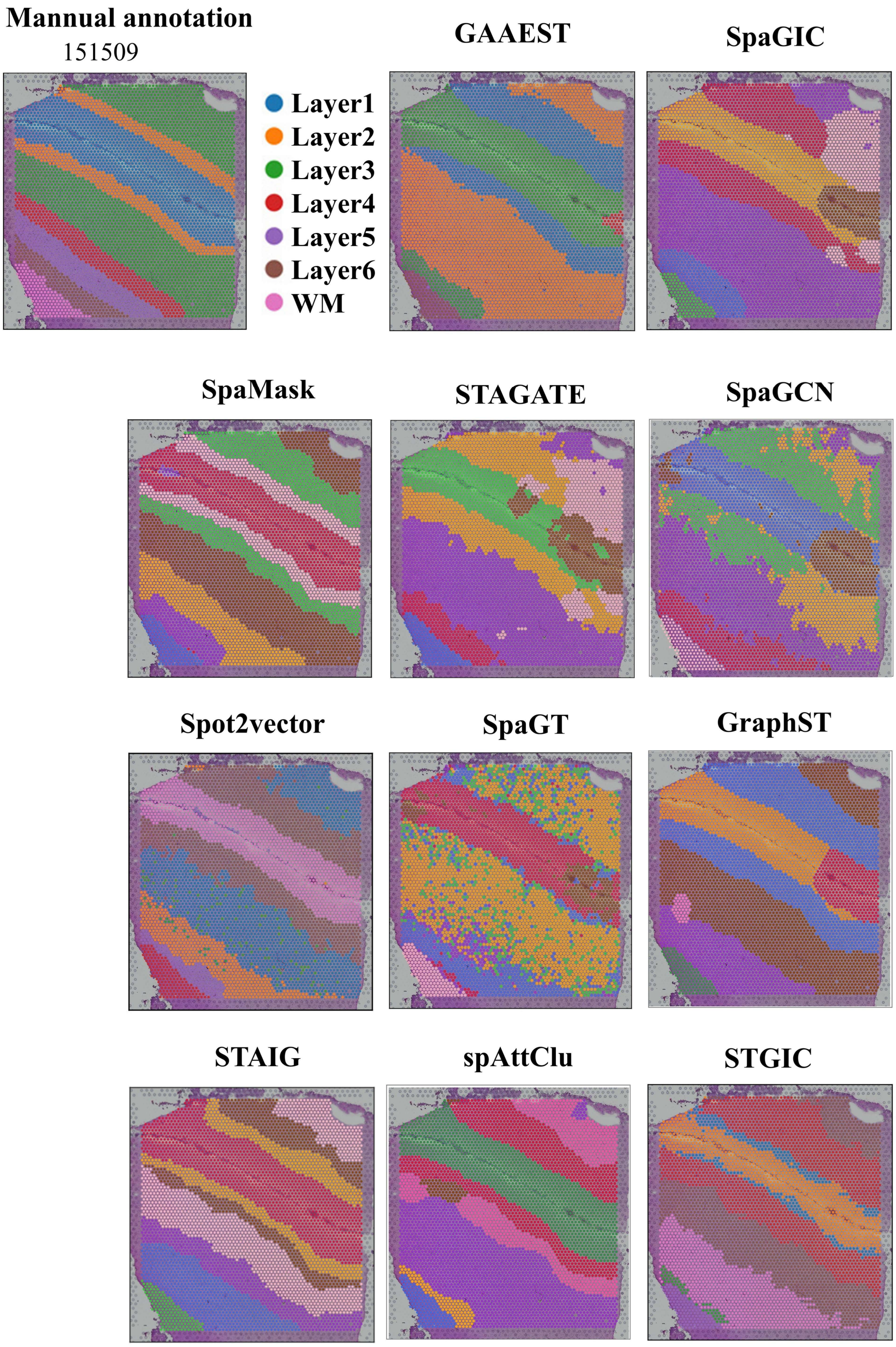

Supplementary S12. This section presents spatial domain clustering results on the DLPFC151510 slice of the DLPFC dataset. The spatial domains output by the proposed model exhibits smooth and coherent band-like structures without scattered noise interference.

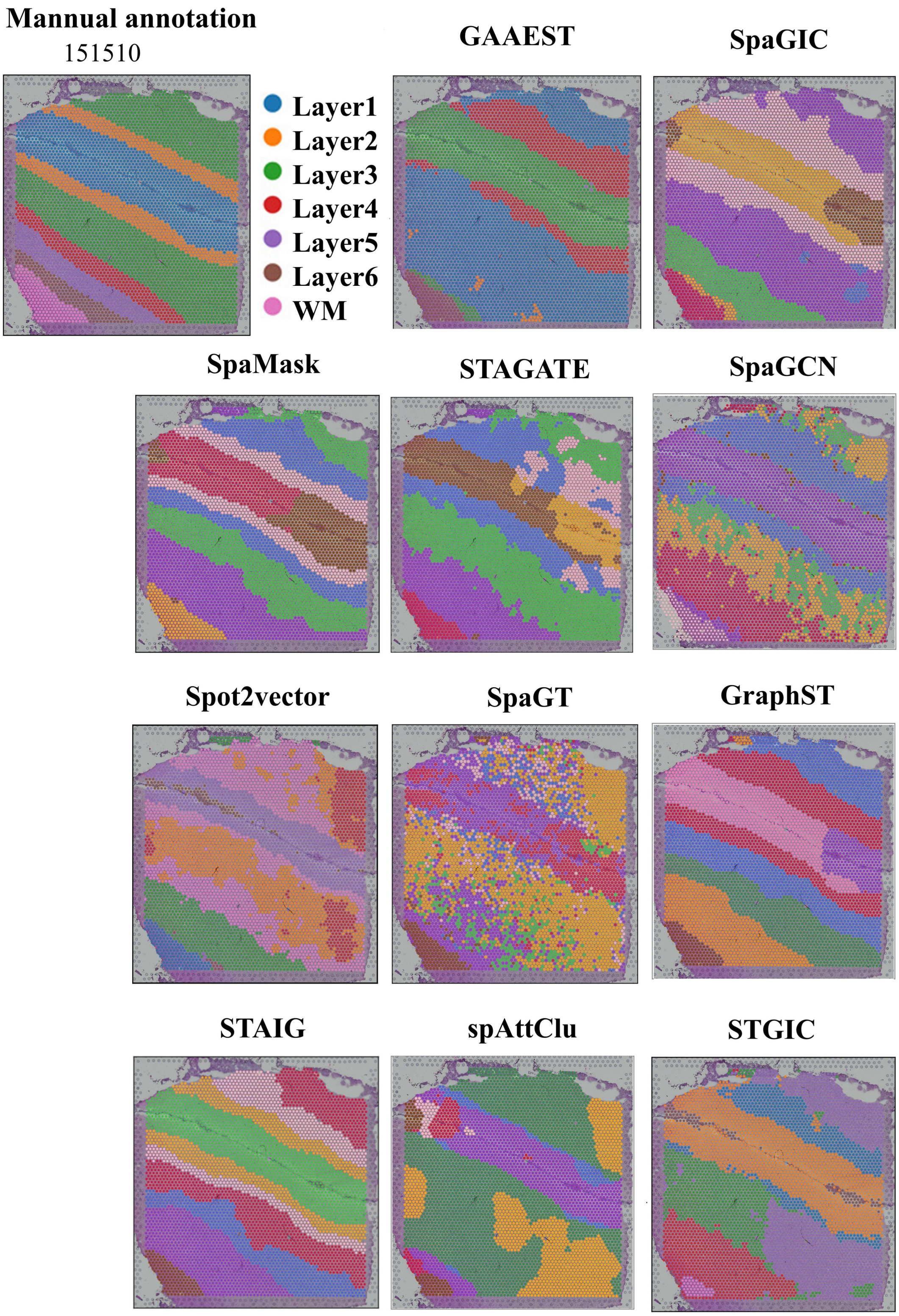

Supplementary S13. This section presents spatial domain clustering results on the DLPFC151669 slice of the DLPFC dataset. The proposed model achieves clear delineation of boundaries between layers.

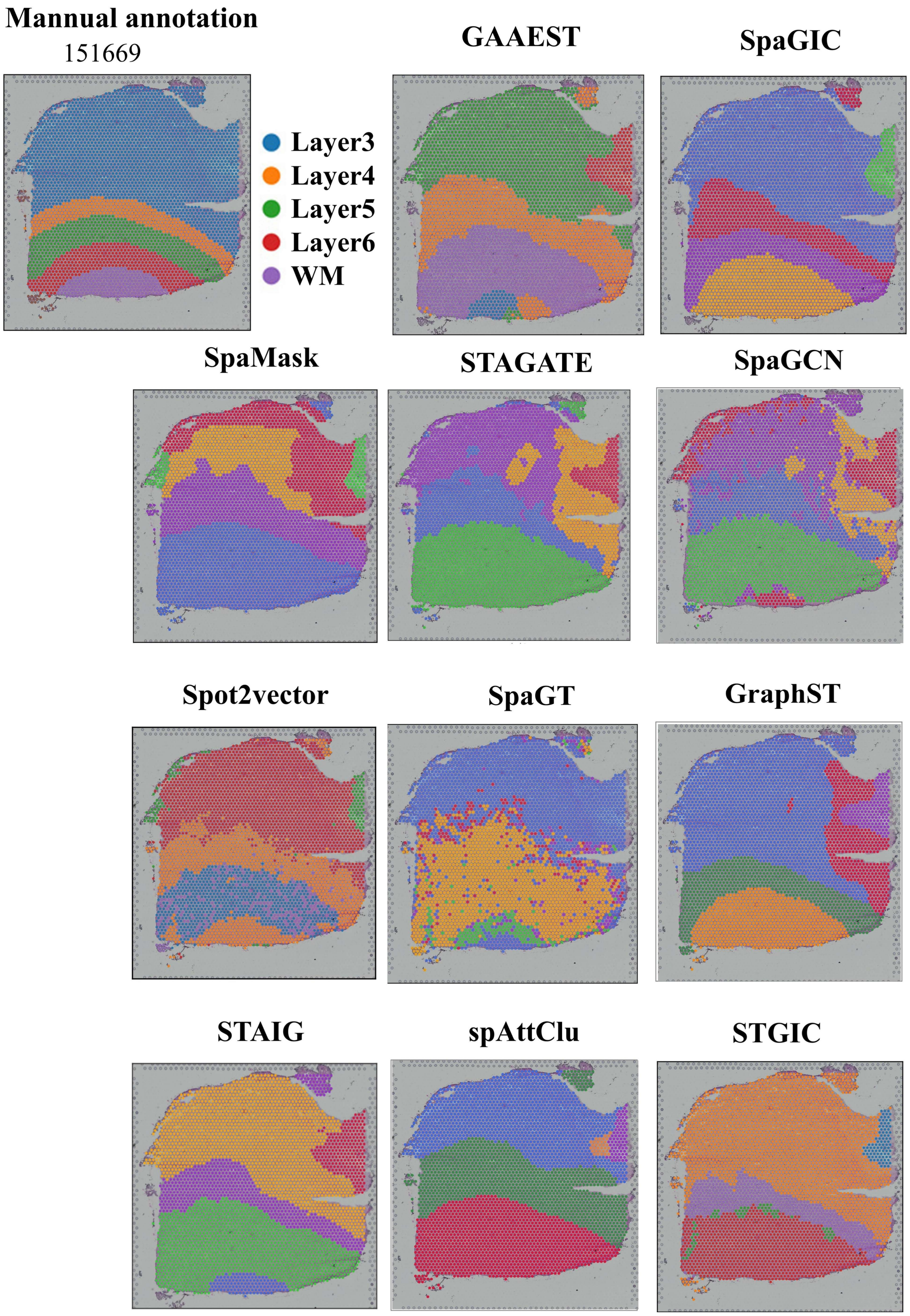

Supplementary S14. This section presents spatial domain clustering results on the DLPFC151670 slice of the DLPFC dataset. The boundaries between layers delineated by the proposed model are coherent and smooth without fragmentation.

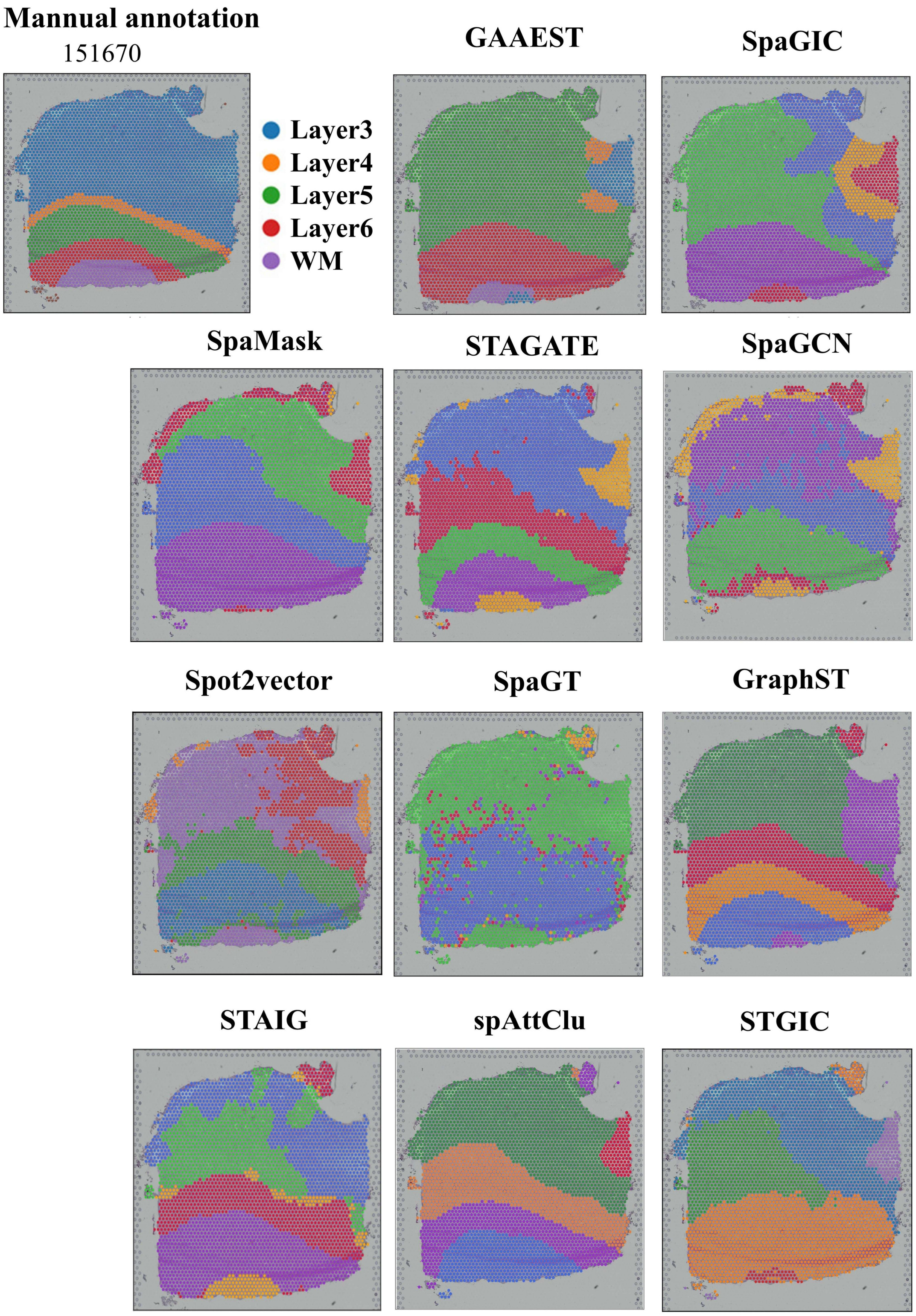

Supplementary S15. This section presents spatial domain clustering results on the DLPFC151671 slice of the DLPFC dataset. The proposed model achieves high accuracy in thickness delineation of Layer 6.

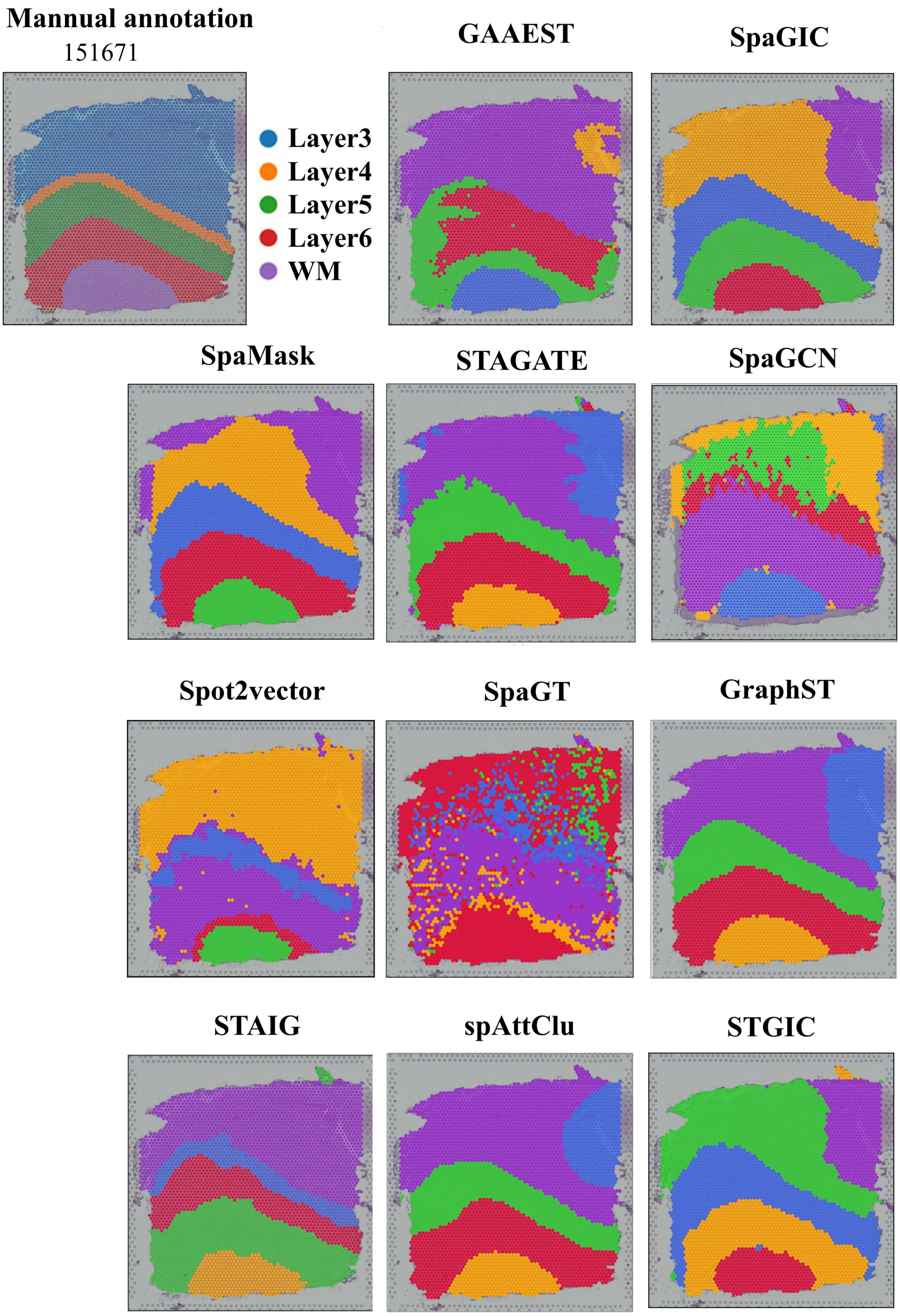

Supplementary S16. This section presents spatial domain clustering results on the DLPFC151672 slice of the DLPFC dataset. The proposed model generates smooth boundaries of Layer 5 with precise thickness delineation.

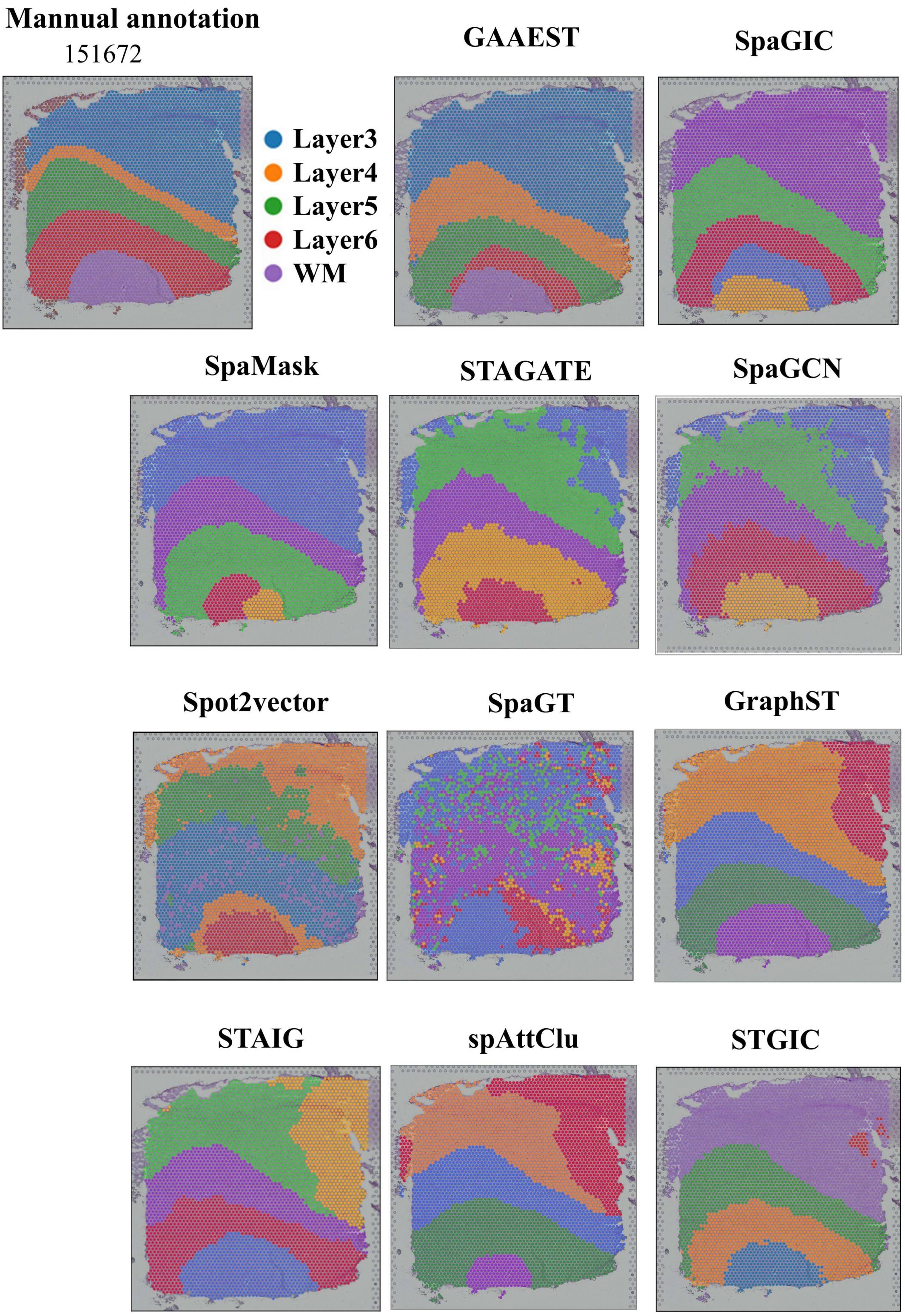

Supplementary S17. This section presents spatial domain clustering results on the DLPFC151673 slice of the DLPFC dataset. The proposed spAttClu model demonstrates the ability to achieve clear delineation of boundaries between layers.

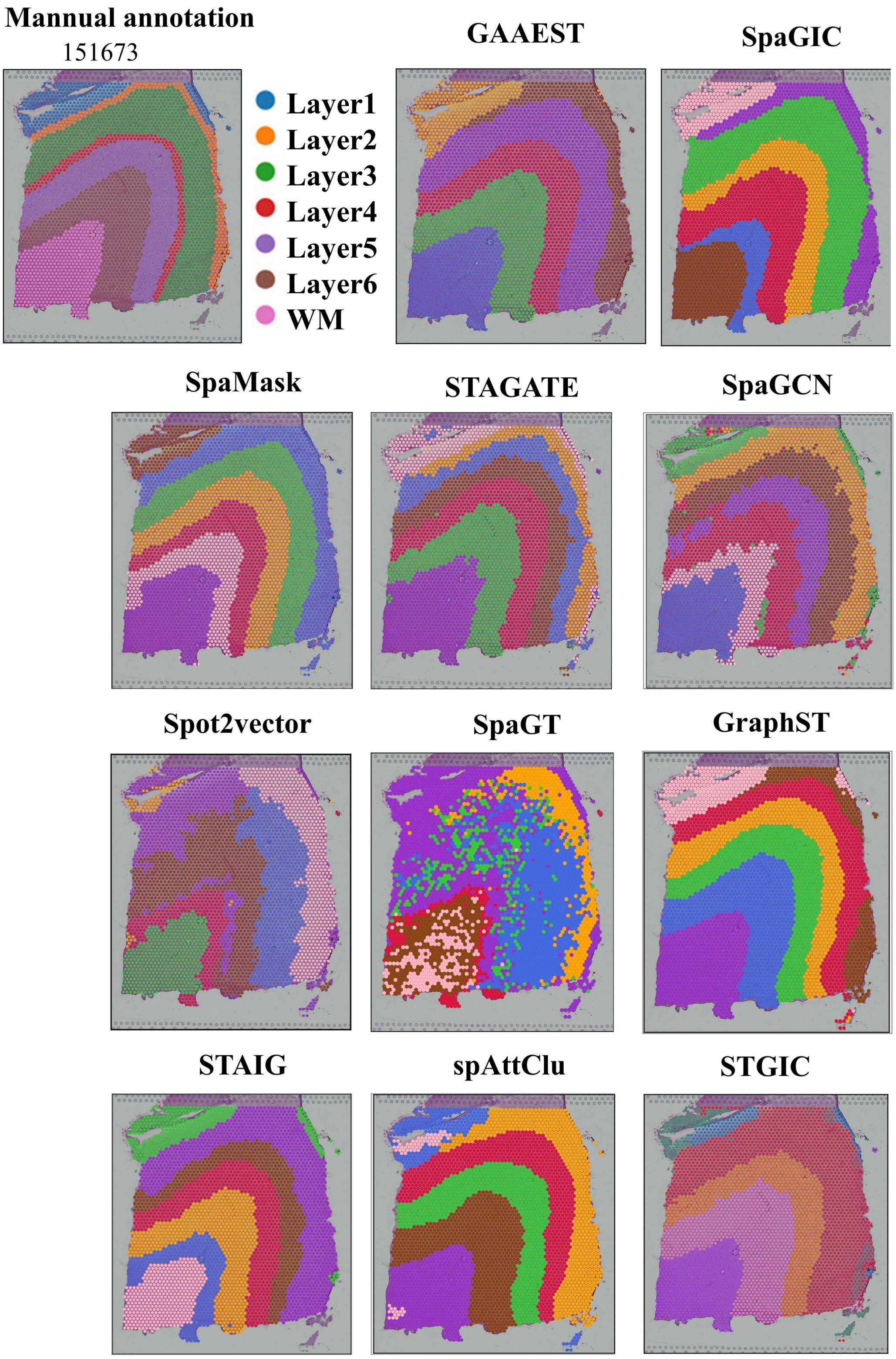

Supplementary S18. This section presents spatial domain clustering results on the DLPFC151674 slice of the DLPFC dataset. The proposed model produces spatial partitioning of Layer 1 without spurious small patches and with accurate thickness delineation.

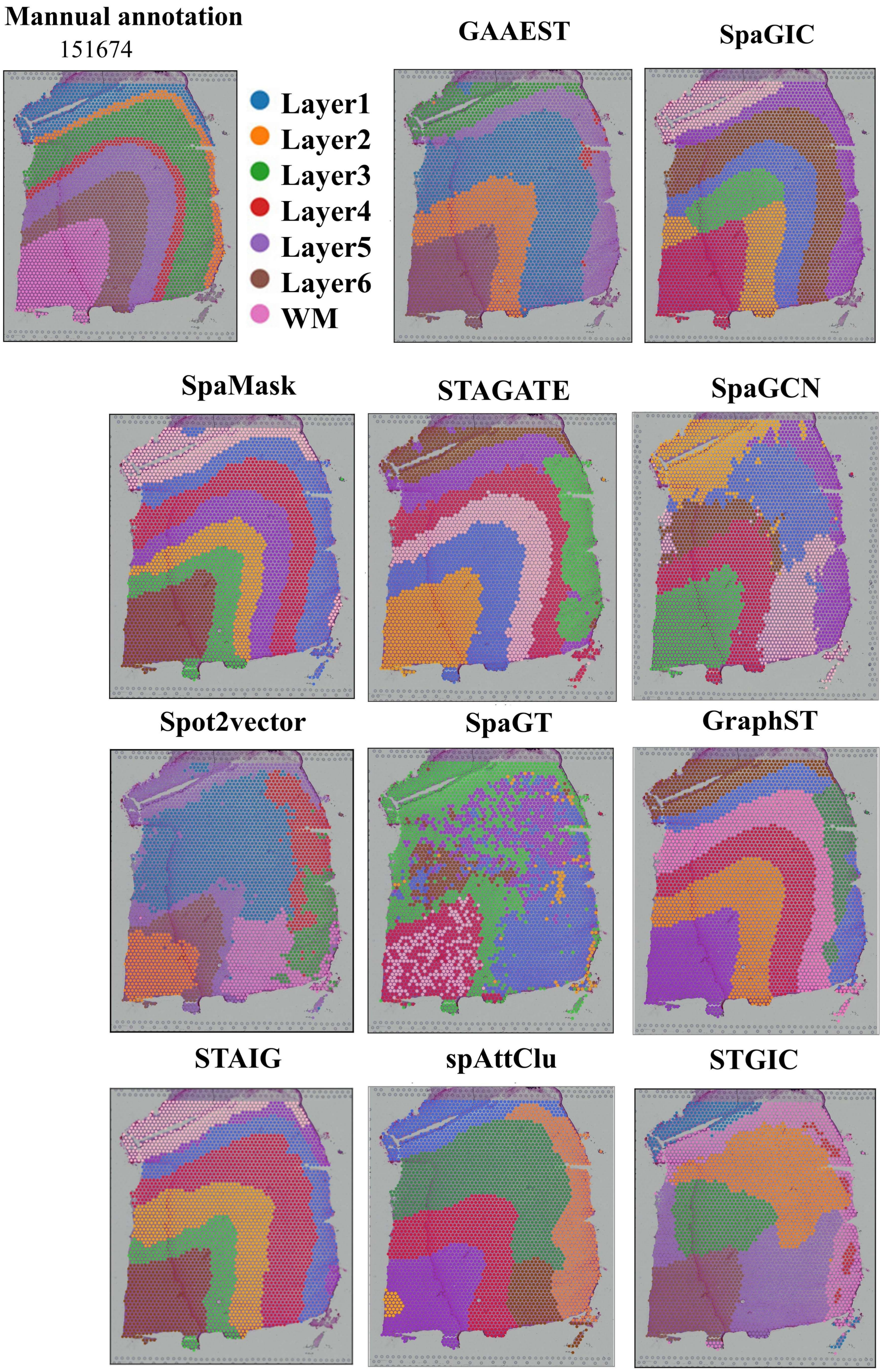

Supplementary S19. This section presents spatial domain clustering results on the DLPFC151675 slice of the DLPFC dataset. The spatial domains output by the proposed model are continuous, large, and smooth regions.

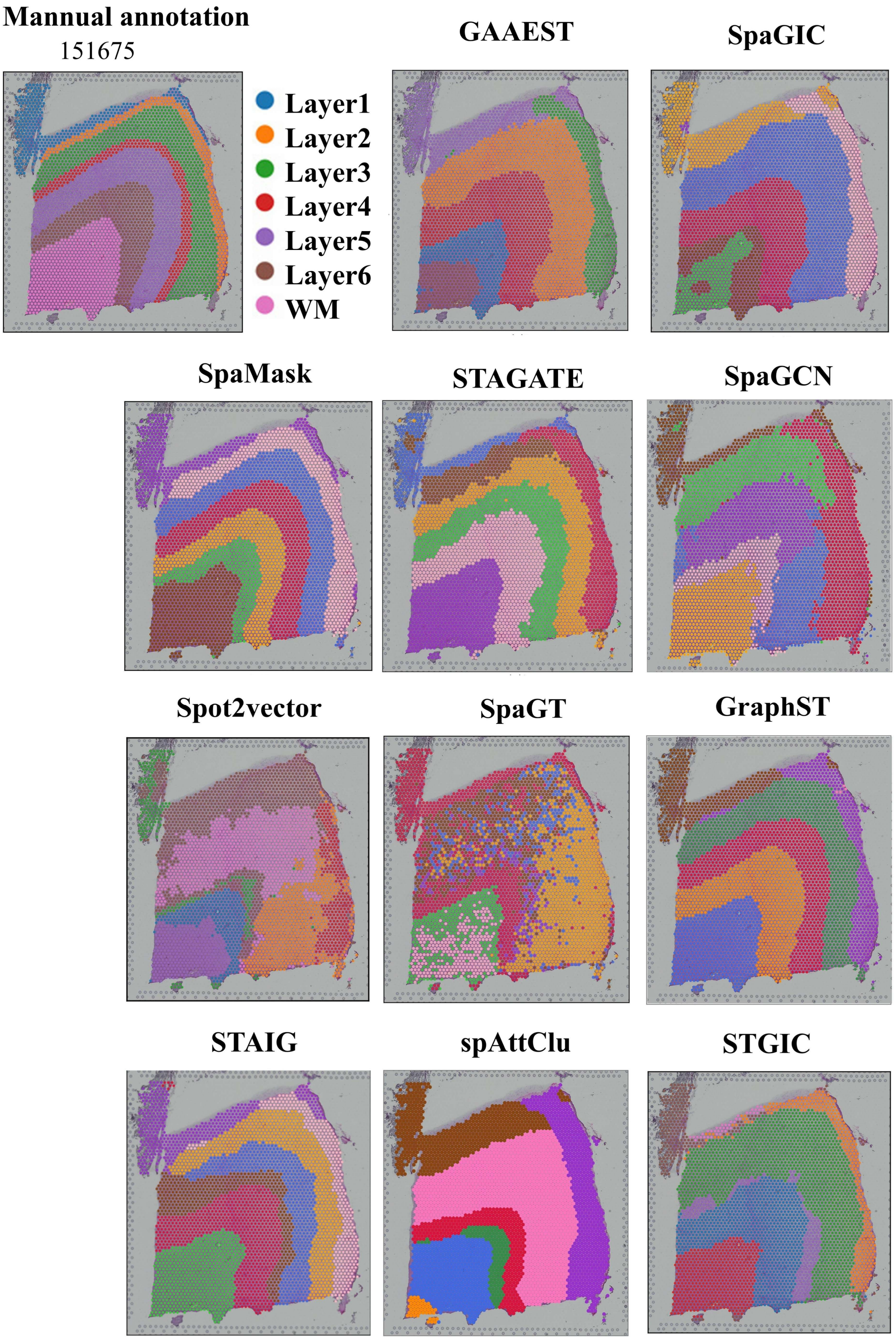

Supplementary S20. This section presents spatial domain clustering results on the DLPFC151676 slice of the DLPFC dataset. The region boundaries delineated by the proposed model are coherent and smooth.

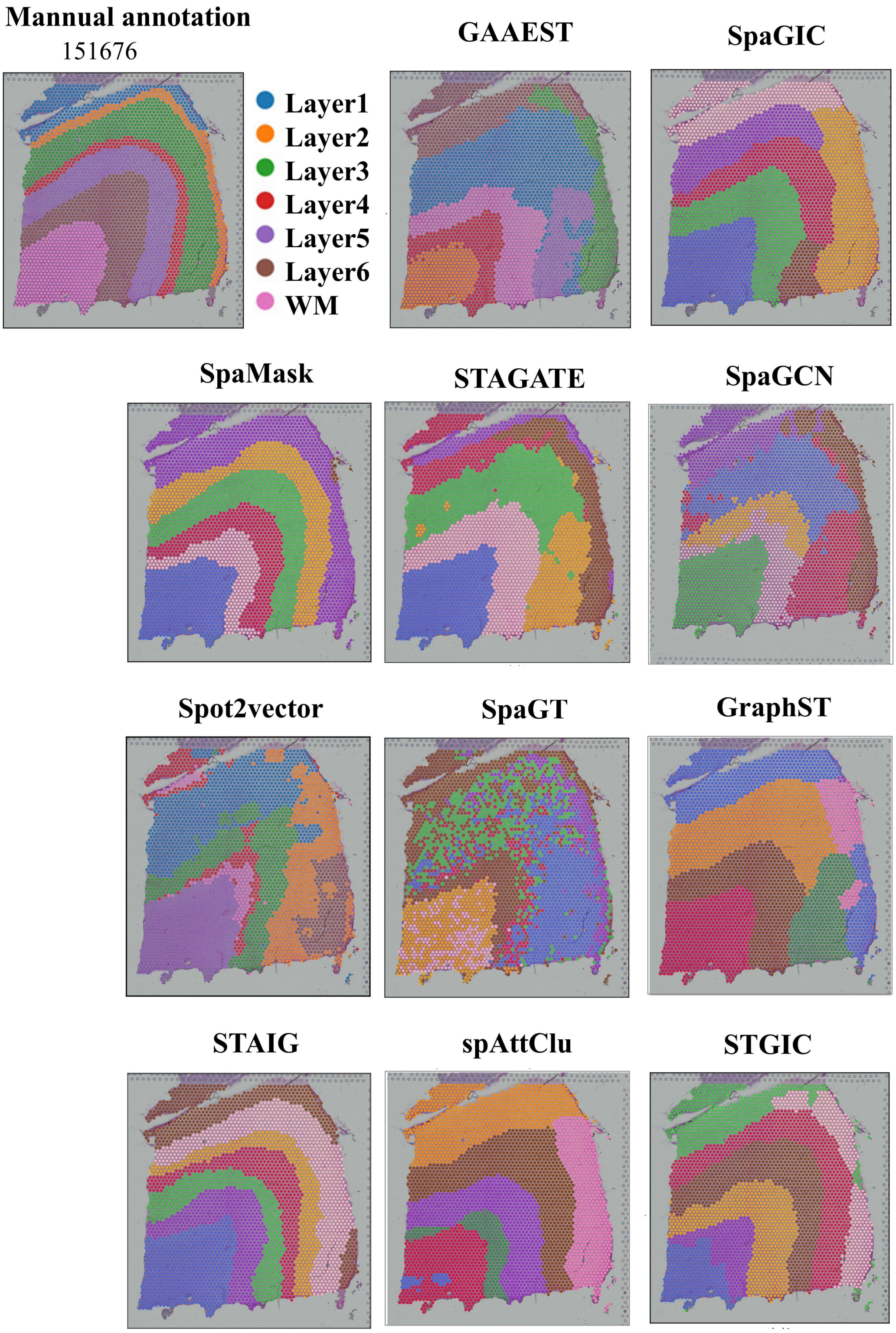

Supplementary S21. This section presents quantitative clustering results on four DLPFC slices (151507, 151508, 151509, 151510). The proposed spAttClu model achieves high ARI and NMI values across all these slices, demonstrating favorable clustering performance.

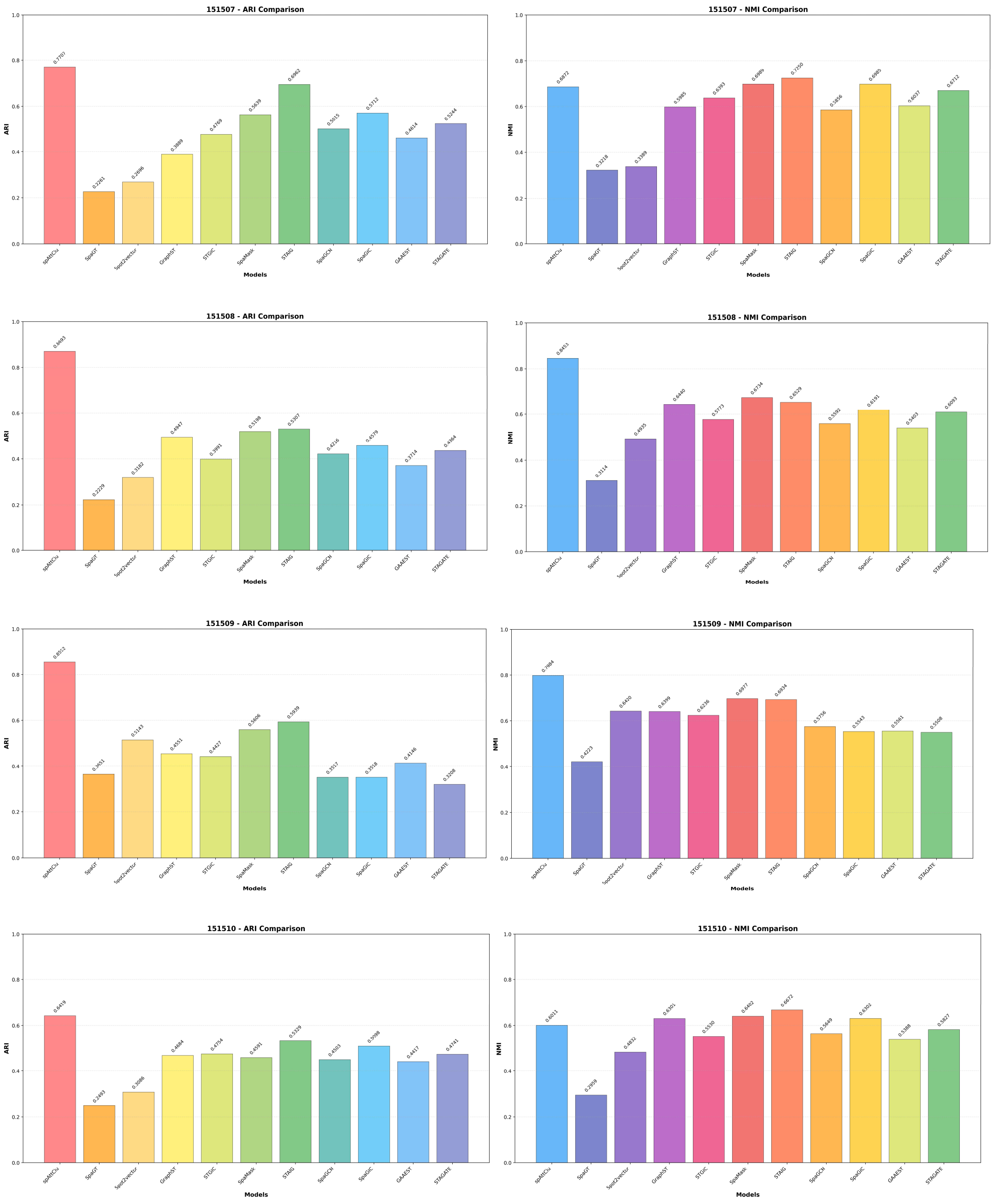

Supplementary S22. This section presents quantitative clustering results on four DLPFC slices (151669, 151670, 151671, 151672). The proposed spAttClu model achieves high ARI and

NMI values across all these slices, demonstrating favorable clustering performance.

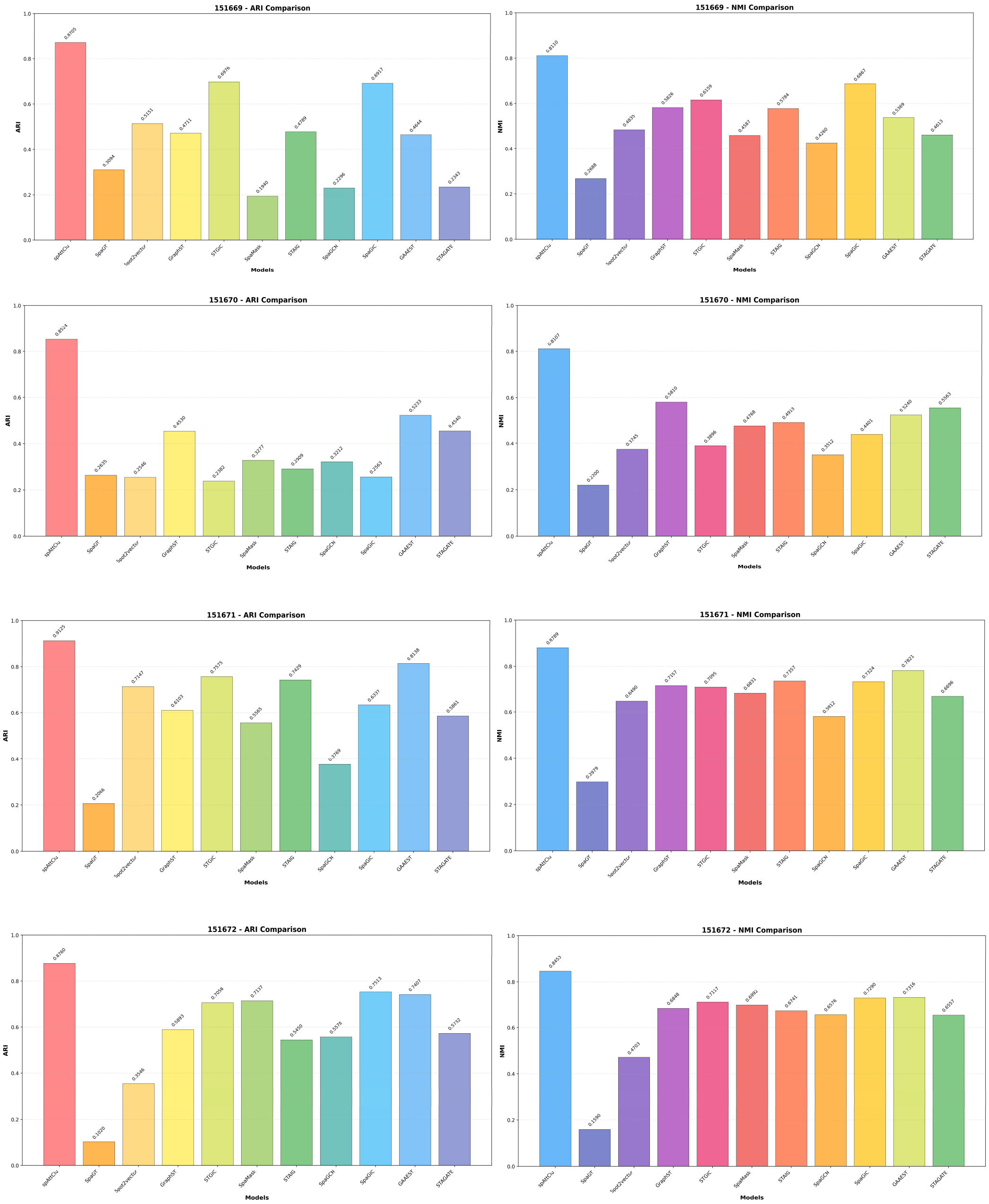

Supplementary S23. This section presents quantitative clustering results on four DLPFC slices (151673, 151674, 151675, 151676). The proposed spAttClu model achieves high ARI and NMI values across all these slices, demonstrating favorable clustering performance.

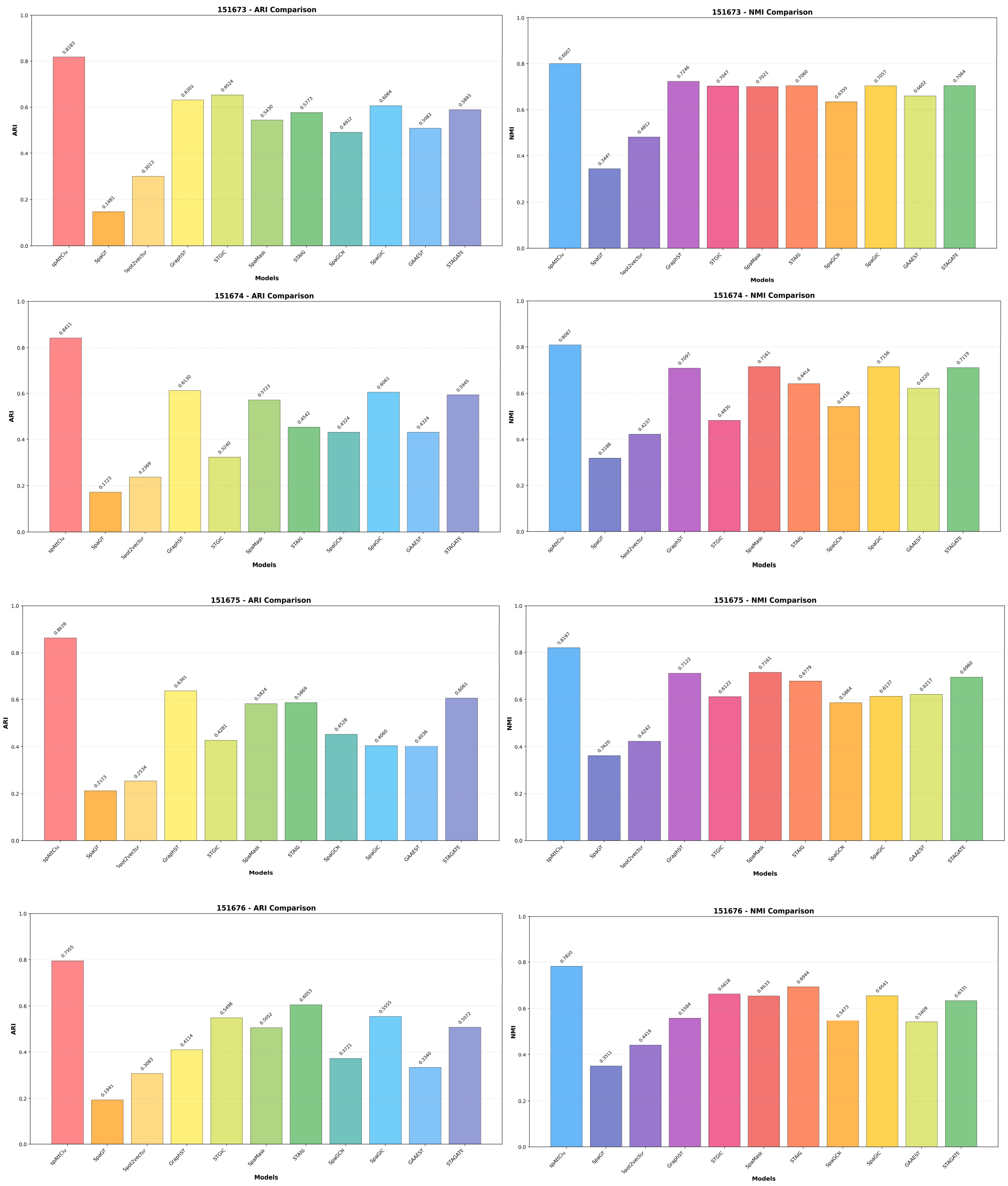

Supplementary S24. The figure below shows the expression distribution of the top 10 SVGs detected in each layer of the DLPFC 151673 slice, highlighting important spatial domain-related GO terms verified by GO analysis with high confidence (Adjusted P-value < 0.05)(Bai, et al., 2025). As shown, Cluster1 is significantly enriched in synaptic signaling and cell-cell signaling, with marker genes including myelin-associated genes MBP and PLP1, suggesting that this region participates in neuronal communication during myelination (Maynard, et al., 2021). Cluster3 is enriched in cytoskeleton organization and actin filament-based processes, and combined with high

expression of ACTA2, TAGLN, and VIM, indicates a role in vascular/stromal structural support (Maynard, et al., 2021). Cluster5 shows multiple neuron development-related terms, including axon development, neuron development, and cell morphogenesis, with high expression of NEFL, NEFM, and NEFH, confirming it as a neuronal projection region (Maynard, et al., 2021). Cluster4 is significantly enriched in positive regulation of cell differentiation, suggesting a potential role in promoting cell differentiation (Maynard, et al., 2021). These results demonstrate that the spatial domains identified by our model are not only statistically significant in expression patterns but also gain biological interpretability through GO functional enrichment, validating the model's effectiveness in revealing tissue spatial heterogeneity and its underlying biological processes.

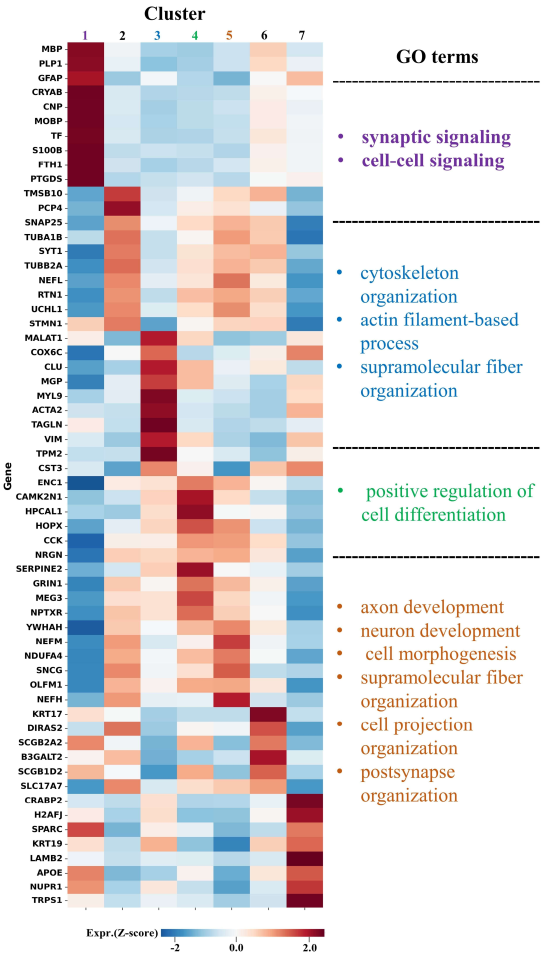

Supplementary S25. For vertical integration of the DLPFC dataset, the proposed model first

loads all consecutive slices, screens highly variable genes (HVGs) by calculating their co-occurrence frequency across slices, and selects HVGs identified in at least two slices as the unified feature set. Subsequently, all slices are concatenated vertically and batch effects across slices are corrected using ComBat (Pratama, et al., 2025). Next, a KNN adjacency matrix is constructed based on spatial coordinates within each slice, and the adjacency matrices of individual slices are concatenated along the diagonal to form a global block-diagonal matrix, enabling vertical integration without cross-slice connections while preserving within-slice spatial structures. The model trains on the integrated expression matrix using a graph attention autoencoder, with a loss function comprising feature reconstruction loss (weight $\alpha = 10$), contrastive learning loss ($\beta = 1$), and spatial regularization loss ($\gamma = 0.3$). The spatial loss uses the attention weights output by the decoder to perform a weighted summation of latent representation differences between neighboring points, encouraging spatially adjacent points to have similar low-dimensional embeddings. After training, latent representations are extracted per slice, followed respectively by mclust clustering and neighborhood refinement (radius = 50), achieving vertical integration and spatial domain identification of consecutive slices.

For horizontal integration of mouse forebrain and hindbrain slices, the proposed model first aligns the spatial coordinates of hindbrain slices to the forebrain reference coordinate system via boundary scaling and translation, then constructs a cross-slice joint neighborhood graph based on the aligned spatial positions to form an undirected joint adjacency matrix. Subsequently, a graph attention network encoder-decoder architecture is employed for joint embedding learning, with key parameters set as embedding dimension 64, training epochs 600, loss weights $\alpha = 10$, $\beta = 1$, and $\gamma = 0.1$. Finally, the integrated low-dimensional representation is output, thereby achieving

biological horizontal integration of spatial transcriptomic data across different slices.

Supplementary S26. Running time of different methods on the E10.5 dataset.

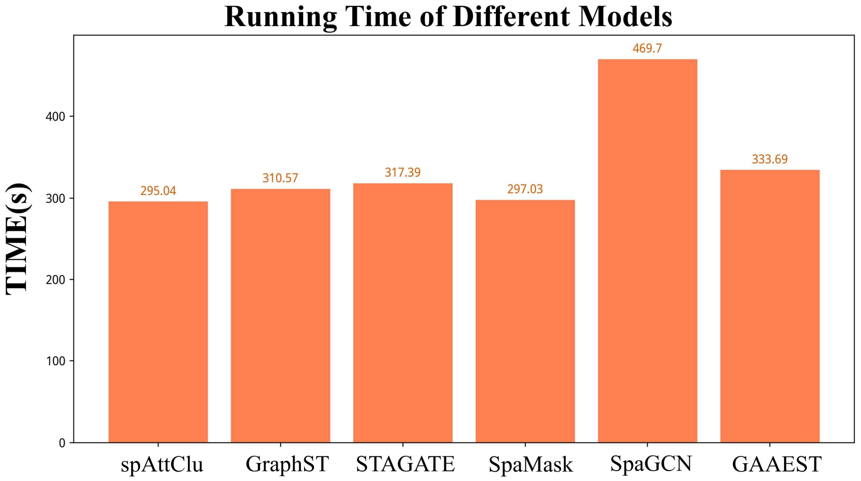

Supplementary S27. To further evaluate model scalability on larger-scale data, we conducted additional clustering experiments on an ultra-large-scale simulated dataset containing 640,000 spots, with all comparison methods using a unified batch size of 15,000 to ensure fair testing. Experimental results show that the proposed model successfully performs clustering on this dataset. In terms of resource consumption, our method uses only 1357 MB of memory, significantly lower than STAGATE's 3277.84 MB. In runtime efficiency, our method demonstrates faster computation speed (6.57 hours). In clustering performance, our model achieves an ARI of 0.4766, approximately 11.9% higher than the second-best method (STAGATE, 0.4259). These findings preliminarily support the effective operation capability of our model on ultra-large-scale data.

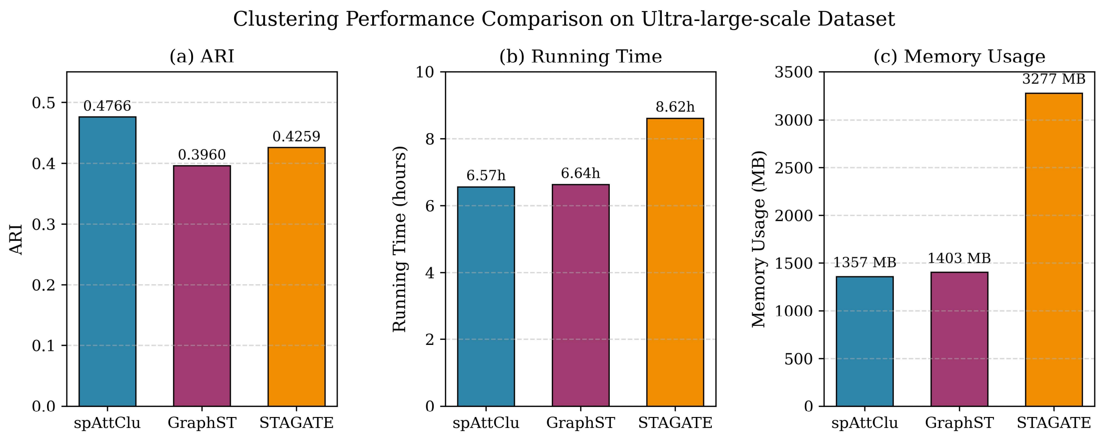
Clustering Performance Comparison on Ultra-large-scale Dataset
(a) ARI
ARI
0.4766
0.3960
0.4259
spAttClu
GraphST
STAGATE
(b) Running Time
Running Time (hours)
6.57h
6.64h
8.62h
spAttClu
GraphST
STAGATE
(c) Memory Usage
Memory Usage (MB)
1357 MB
1403 MB
3277 MB
spAttClu
GraphST
STAGATE

Supplementary Table S1. Consolidated hyperparameter configuration

Category	Parameter	Value
**Preprocessing**	n_top_genes	3000
**Preprocessing**	K (10x Visium)	3
**Preprocessing**	K (STARmap)	3
**Preprocessing**	K (osmFISH)	3
**Preprocessing**	K (MERFISH)	3
**Preprocessing**	K (Stereo-seq standard)	4
**Preprocessing**	K (Stereo-seq large-scale)	3
**Model architecture**	input_dim	3000
**Model architecture**	hidden_dim (GAT output)	64
**Model architecture**	latent_dim	64
**Model architecture**	encoder_sc hidden layers (Encoder_scRNA)	[256, 64, 32]
**Training**	epochs	600
**Training**	learning_rate (ST)	0.001
**Training**	learning_rate (scRNA)	0.01
**Training**	batch_size	15000
**Training**	optimizer	Adam
**Loss weights**	alpha	10
**Loss weights**	beta	1
**Loss weights**	gamma	0.1
**Clustering**	method (default)	mclust
**Reproducibility**	random_seed	41

Supplementary Table S2. Wilcoxon signed-rank test p-values between the proposed model and comparison methods on 12 slices of the DLPFC dataset.

Model	spaGT	spot2vector	graphST	STGIC	SpaMask	staig	spaGCN	spaGIC	GAAEST	stagate
**ARI**	2.44E-04	2.44E-04	2.44E-04	2.44E-04	2.44E-04	2.44E-04	2.44E-04	2.44E-04	2.44E-04	2.44E-04
**NMI**	2.44E-04	2.44E-04	4.88E-04	2.44E-04	1.22E-03	1.22E-03	2.44E-04	1.22E-03	2.44E-04	2.44E-04

References

Bai, Y., *et al.* SpaSEG: unsupervised deep learning for multi-task analysis of spatially resolved transcriptomics. *Genome Biol* 2025;26(1).
Gong, Y.Q., Yuan, X., Jiao, Q. and Yu, Z.S. Unveiling fine-scale spatial structures and amplifying gene expression signals in ultra-large ST slices with HERGAST. *Nat Commun* 2025;16(1).
Maynard, K.R., *et al.* Transcriptome-scale spatial gene expression in the human dorsolateral prefrontal cortex. *Nat Neurosci* 2021;24(3):425-436.
Min, W.W., Fang, D.H., Chen, J.Y. and Zhang, S.H. SpaMask: Dual masking graph autoencoder with contrastive learning for spatial transcriptomics. *Plos Comput Biol* 2025;21(4).
Pratama, R., Hilton, J., Cherry, J.M. and Song, G. Gene spatial integration: enhancing spatial transcriptomics analysis via deep learning and batch effect mitigation. *Bioinformatics* 2025;41(6).
Wang, Y., *et al.* mNSF: multi-sample non-negative spatial factorization. *Genome Biol* 2025;26(1).
Yang, Y.T., *et al.* STAIG: Spatial transcriptomics analysis via image-aided graph contrastive learning for domain exploration and alignment-free integration. *Nat Commun* 2025;16(1).
